# The transcription factor WRKY25 can act as redox switch to drive the expression of *WRKY53* during leaf senescence in Arabidopsis

**DOI:** 10.1038/s41598-025-13023-1

**Published:** 2025-07-29

**Authors:** Ana Gabriela Andrade Galan, Jasmin Doll, Edda von Roepenack-Lahaye, Natalie Faiss, Ulrike Zentgraf

**Affiliations:** Center for Plant Molecular Biology (ZMBP), University of Tübingen, Auf der Morgenstelle 32, 72076 Tübingen, Germany

**Keywords:** *Arabidopsis thaliana*, Senescence regulation, WRKY transcription factors, WRKY homo- and heterodimerization, Redox regulation, NOS bridge, Plant development, Plant molecular biology, Plant signalling

## Abstract

Senescence requires high plasticity and, therefore, must be coordinated by a complex regulatory network. Notably, WRKY transcription factors highly impact senescence regulation. WRKYs can form homo- and heterodimers and contain the binding motifs of WRKY factors in their promoters already forming a complex regulatory network between themselves. For the Arabidopsis hub gene *WRKY53*, WRKY18 acts as a strong negative while WRKY25 serves as strong positive regulator, creating a smaller subnetwork with high complexity, which we analyzed in detail. Activation of *WRKY53* expression by WRKY25 is redox sensitive while repression by WRKY18 was not. Deletions and domain-swapping between WRKY18 and WRKY25 revealed that the N-terminal domain of WRKY25 is crucial for its activator effect on *WRKY53* expression. Moreover, WRKY25 does not form homodimers but is able to heterodimerize with WRKY18 also requiring its N-terminal domain. The impact on senescence regulation and on *WRKY53* expression was validated *in planta* using transgenic complementation lines of the *wrky25* mutant. Modeling WRKY25 *in silico* indicated a putative covalent lysine-cysteine NOS redox switch. LC–MS analyses suggest that the NOS bridges really exist. We propose that WRKY25 acts as a redox sensor, balancing the expression and interactions of the WRKY53/WRKY25/WRKY18 network to ensure progressive senescence induction.

## Introduction

In agricultural production, well-timed leaf senescence plays an important role not only for the fitness of the whole plant but also influences crop yield quantity and quality^1–3^. Senescence is the tightly regulated and programmed final stage of plant development. The aim of senescence is to maximize the relocation of vital nutrients such as carbon, nitrogen, and mineral resources out of senescing tissues to developing parts of the plants^4,5^. The age of individual leaves and the age of the whole plant are the main factors driving developmental senescence under normal non-stress conditions. The plant senses these parameters through a multitude of well-coordinated signals that initiate and modulate senescence. It has been widely described that nearly all plant hormones can influence the senescence program, as well as small signaling molecules such as peptides, calcium, and reactive oxygen species (ROS)^6–14^.

At the transcriptional level, several thousand genes are upregulated and downregulated during the onset and progression of senescence in *Arabidopsis thaliana*, leading to extensive reprogramming of the transcriptome and highlighting the crucial role of transcription factors^15–22^. Among these genes, two transcription factor families namely WRKY and NAC factors, are notably overrepresented in the transcriptome of Arabidopsis during senescence^16^. For many factors belonging to these families, a regulatory role has already been characterized across various plant families^21,23,24^. A notable feature of the WRKY family members is the presence of the W-box (TTGAC(C/T)) DNA-binding motif in their own promoters. This motif allows WRKY transcription factors to regulate each other, forming a complex WRKY-driven transcriptional network^25^.

In *Arabidopsis thaliana*, WRKY53 has been characterized as a positive regulator of developmental senescence and functions as one of the key regulatory hubs involved in several senescence-associated processes such as remobilization, nutrient transport, ROS signaling, and the degradation of ROS molecules^6,10,11,26–28^. Expression and activity as well as degradation of WRKY53 are tightly regulated involving many feedback controls including even several double bottoms. In addition, WRKY53 is involved in epigenetic control of other senescence regulators (for review see^28^).

Among the WRKYs expressed in mature green leaf tissue, WRKY18 and WRKY25 have been identified as most effective repressors and activators of *WRKY53* expression, respectively. WRKY18 serves as a negative upstream regulator, a downstream target, and a protein interaction partner of WRKY53^29^. In contrast, WRKY25 acts as a positive upstream regulator, but also as downstream target and protein interaction partner of WRKY53^13^. Moreover, plant lines with altered expression of these two WRKYs exhibit altered senescence-associated phenotypes. Plants lacking *WRKY18* expression show accelerated senescence, consistent with its role as a repressor of *WRKY53*^29^. Contradictory with its role as an activator of *WRKY53* expression, *wrky25* mutants also show accelerated senescence^13^. Therefore, regulation appears to be more complex and is most likely organized through a small, yet intricate, subnetwork, in which the loss of WRKY25 creates an imbalance leading to this contradictory phenotype. The molecular mechanisms governing interactions within the WRKY18/WRKY25/WRKY53 network and their correlation with leaf senescence remain elusive. Furthermore, a signaling molecule modulating this subnetwork has not yet been characterized in detail. Interestingly, the WRKY25 DNA-binding activity depends on the redox conditions^13^.

Here, we could show that WRKY25 can act as a redox switch, balancing the expression and interactions of the WRKY18/WRKY25/WRKY53 subnetwork to ensure the progressive induction of senescence in *Arabidopsis thaliana*. In addition, the heterodimer of WRKY18 and WRKY25 was identified as an activator of *WRKY53* expression. Dissecting the protein structures by means of deletion constructs and domain-swapping between WRKY18 and WRKY25 revealed that the N-terminus of WRKY25 is crucial for its activator effect on *WRKY53* expression and its ability to heterodimerize. Redox conditions were identified to be critical for regulatory effects and a putative redox switch was discovered in the WRKY25 protein. This study enhances our understanding of plant senescence regulation and highlights the significant role of redox conditions in plant regulatory processes.

## Results

### Transactivation potential with the WRKY53/WRKY25/WRKY18 subnetwork

**Fig. 1:**
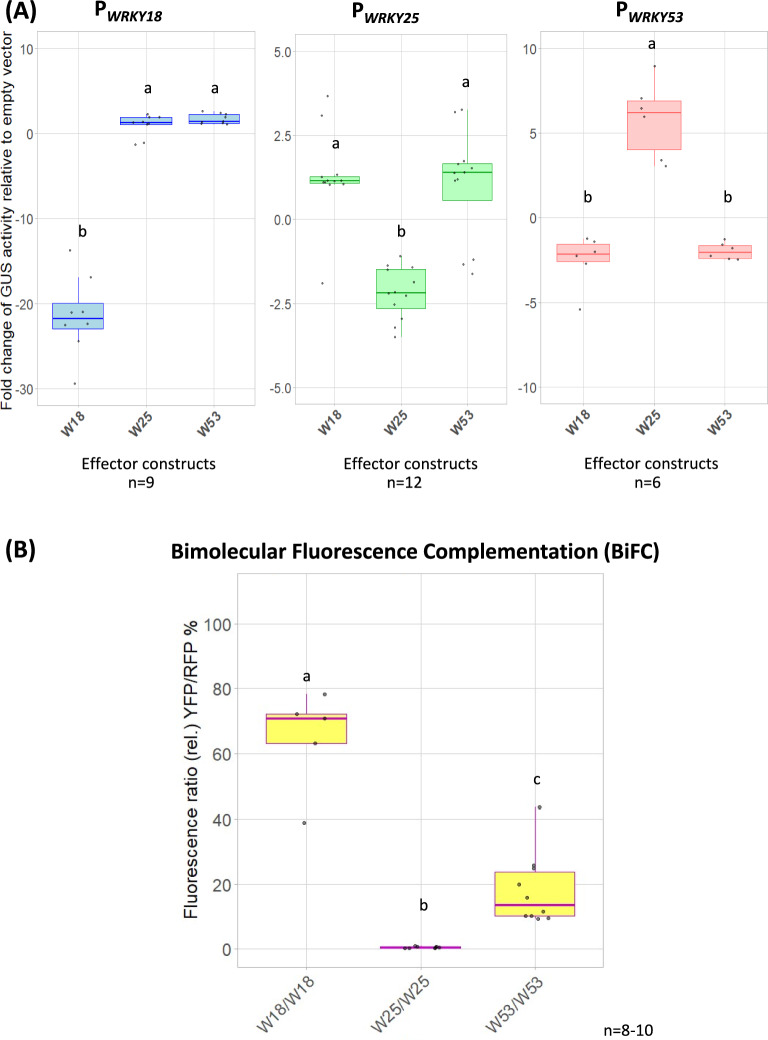
Transactivation assays in Arabidopsis protoplasts for P_*WRKY18*_, P_*WRKY25*_, P_*WRKY53*_, and *in planta* homodimerization of WRKY18, WRKY25, and WRKY53 in *Nicotiana benthamiana*. **(A)** Arabidopsis root protoplasts were transiently transformed with fragments of the promoters of *WRKY18* (3000 bp), *WRKY25* (3000 bp), *and WRKY53* (2759 bp), each fused to the *GUS* reporter gene, along with 35S:*WRKY18* (W18), 35S:*WRKY25* (W25), or 35S:*WRKY53* (W53) as effector constructs. Relative GUS activity (normalized to values of the empty vector) is shown as boxplots. Sample sizes (n) are indicated for each group within the figure and represent independent biological replicates **(B)** Leaves of *N. benthamiana* were transformed with pBiFCt2in1-NN constructs containing the possible combinations for the homodimerization of the three WRKYs of the subnetwork. These transformed leaves were analyzed under a confocal laser scanning microscope: yellow fluorescence (YFP) indicates interaction (BiFC), red fluorescence (RFP) serves as a transformation control. Boxplots representing the relative fluorescence ratio (%YFP/RFP) are presented. Sample size (n) is indicated in the figure and represents independent biological replicates. In both (**A**) and (**B**) one-way ANOVA followed by Tukey’s HSD post-hoc test was performed to determine statistically significant differences among effectors. Different lowercase letters indicate statistically distinct groups (p ≤ 0.05).

To gain further insight into the potential regulatory subnetwork formed by WRKY18, WRKY25, and WRKY53, we studied the interactions among these three WRKYs and their effects on each other’s expression in more detail. Therefore, *Arabidopsis thaliana* protoplasts were transiently co-transformed and utilized as an *in vivo* transactivation system using reporter gene expression. In this system, reporter constructs containing approx. 3000 bp promoter fragments of *WRKY18*, *WRKY25*, and *WRKY53* in front of the glucuronidase (*GUS*) reporter gene, respectively, were co-transformed with different effector constructs (WRKY18, WRKY25, or WRKY53 under the control of a CaMV 35S promoter, respectively). Based on the measured activity of the GUS enzyme in relation to a co-transformed luciferase control, we confirmed that WRKY18, WRKY25, WRKY53 downregulated the reporter gene expression driven by their own promoters (Fig. 1A). As expected, the *WRKY53* expression was upregulated by the WRKY25 effector protein and downregulated by WRKY18 as effector protein (Fig. 1A). In the same context, *WRKY18* expression could be slightly increased by WRKY25 and WRKY53 effectors while *WRKY25* expression could slightly be activated by WRKY18 and WRKY53 effector proteins (Fig. 1A). This indicates that WRKYs can function as activators and repressors depending on the promoter they are interacting with and that WRKY18 had the strongest repressing effect while WRKY25 had the strongest activating effect.

Given that WRKYs have been previously observed to form dimers, protein–protein interactions were evaluated to identify homodimerization within this subnetwork. To detect these interactions, Bimolecular Fluorescence Complementation (BiFC) assays were performed in Arabidopsis protoplasts combined with cell sorting or in *Nicotiana benthamiana* leaves combined with laser scanning microscopy. To achieve fluorescence by protein–protein interaction, they were transiently transformed with constructs containing either WRKY18, WRKY25, or WRKY53 fused to one half of the yellow fluorescent protein (YFP), paired with the same respective WRKY fused to the other half of the YFP. If homodimers can be formed, both halves of YFP are brought in close proximity and can emit fluorescence. A sequence encoding a red fluorescence protein (RFP) is present in the same vector backbone as transformation and expression control. In both transformation systems, homodimerization of WRKY18 and WRKY53 was observed. In *N. benthamiana* leaves, this was evidenced by the fluorescence intensity ratio (YFP/RFP) calculated from microscopy image measurements. In contrast, WRKY25 did not exhibit homodimerization in either system. (Fig. 1B and S1).

### The heterodimer WRKY18-WRKY25 is an activator of WRKY53 expression

**Fig. 2:**
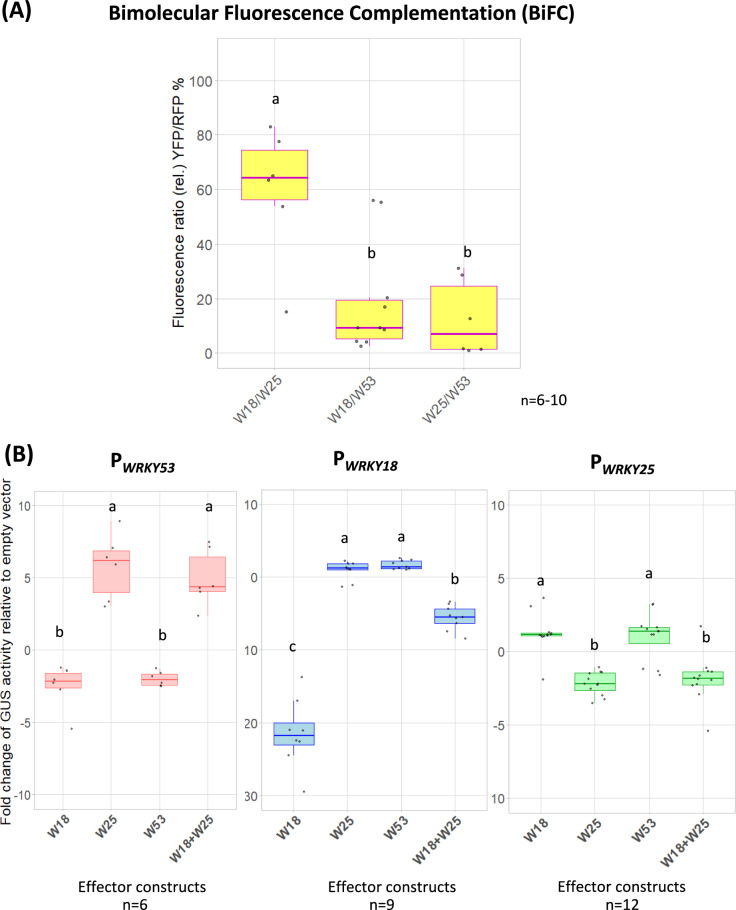
*In planta* heterodimerization of WRKY18, WRKY25, and WRKY53 in *N. benthamiana*, and transactivation assays in Arabidopsis protoplasts for the effect of WRKY18/WRKY25 heterodimer effect on the P_*WRKY53*_*.* **(A)** Leaves of *N. benthamiana* were transformed with pBiFCt2in1-NN constructs containing the possible combinations for the heterodimerization of the three WRKYs of the subnetwork. The transformed leaves were analyzed under a confocal laser scanning microscope: yellow fluorescence (YFP) indicates interaction (BiFC), and red fluorescence (RFP) serves as a transformation control. Boxplots representing the relative fluorescence ratio (%YFP/RFP) are presented. Sample size (n) is indicated in the figure and represents independent biological replicates. **(B)** Arabidopsis protoplasts were transformed with fragments of the promoters of *WRKY18* (3000 bp), *WRKY25* (3000 bp), and *WRKY53* (2759 bp), each fused to the *GUS* reporter gene, along with 35S:*WRKY18* (W18), 35S:*WRKY25* (W25), 35S:*WRKY53* (W53), or 35S:*WRKY18/WRKY25* (W18 + W25) as effector constructs. Values relative to values of the empty vector control are presented as boxplots, with sample sizes (n) shown for each group within the plot representing independent biological replicates. In both (**A**) and (**B**) one-way ANOVA followed by Tukey’s HSD post-hoc test was performed. Lowercase letters indicate statistically significant differences between groups (p ≤ 0.05).

To address the question whether these WRKYs could also form heterodimers and what impact this would have on the subnetwork, we examined the potential heterodimerization, as heterodimer formation within the WRKY family has been widely documented^30–32^. Using the BiFC system in *N. benthamiana* leaves, it was possible to identify interactions among all three WRKYs, however the WRKY18/WRKY25 heterodimer stood out above the others based on a stronger YFP signal under the confocal microscope and a higher intensity calculated from the YFP/RFP ratio (Fig. 2A and S2). Therefore, we were curious what would be the consequences of the WRKY18/WRKY25 heterodimer formation within the subnetwork. Testing WRKY18/WRKY25 heterodimer in our in vivo transactivation system on the different promoters revealed that only the expression of the reporter gene driven by the *WRKY53* promoter was strongly upregulated by the heterodimer (Fig. 2B) indicating that the heterodimer behaves more similar to WRKY25. Consistently, the heterodimer downregulated the *GUS* expression driven by the promoter of *WRKY25* (Fig. 2B) whereas the heterodimer was less efficient in inhibiting the reporter gene expression by the *WRKY18* promoter also suggesting that the heterodimer acts more like WRKY25 alone (Fig. 2B). These results demonstrate that WRKY25 and WRKY18 can heterodimerize and that this heterodimer significantly influences the subnetwork’s activity.

### The N-terminus of WRKY25 is necessary for the activation of the WRKY53 and WRKY18 promoters while the C-terminus interferes with dimerization

**Fig. 3:**
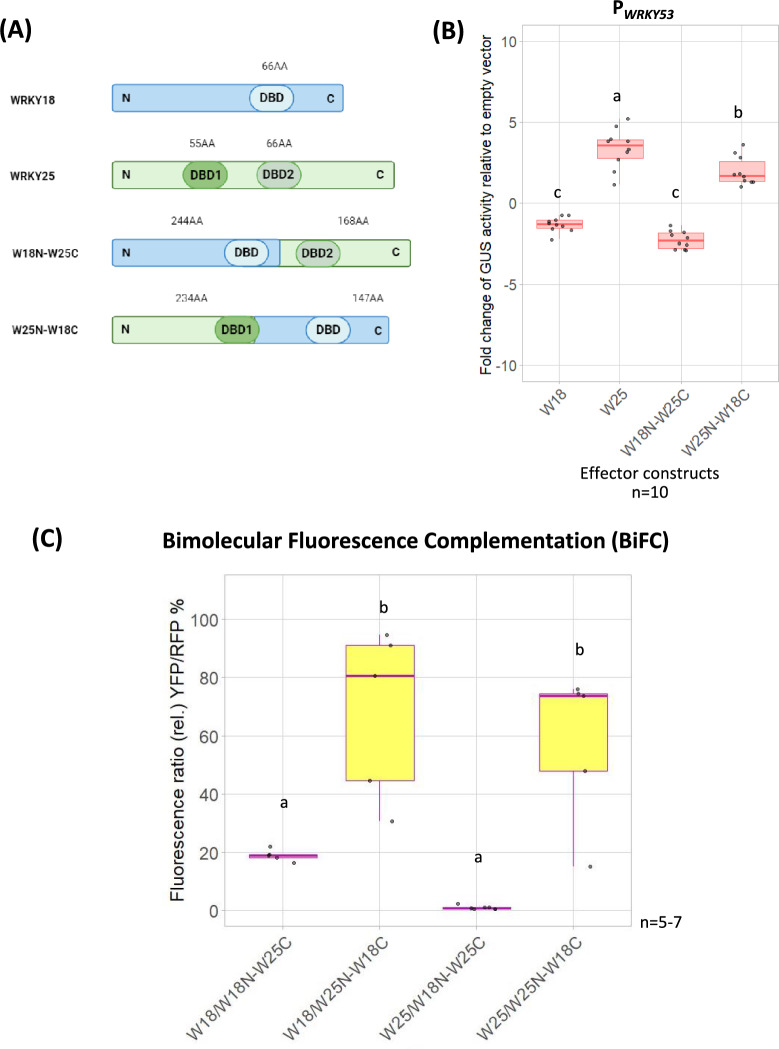
Transactivation assay in Arabidopsis protoplasts for the P_*WRKY53*_ and the effect of the chimeras on it, and *in planta *protein–protein interactions of WRKY18 and WRKY25 with the chimeras in *N. benthamiana*. **(A)** Schematic drawing represents the native WRKY18 and WRKY25 protein as well as the chimeras between these WRKYs: W18N-W25C and W25N-W18C. **(B)** Arabidopsis root protoplasts were transiently transformed with the fragment of the*WRKY53* promoter (2759 bp), fused to the *GUS* gene as a reporter construct, along with 35S:*WRKY18* (W18), 35S:*WRKY25* (W25), 35S:*W18N-W25C*, or 35S:*W25N-W18C* as effector constructs. GUS activity values, expressed relative to the values of the empty vector, are shown as boxplots. Sample size (n = 10) corresponds to independent biological replicates. **(C)** Leaves of *N. benthamiana* were transformed with pBiFCt2in1-NN constructs containing the possible combinations for the interactions between WRKY18 or WRKY25 with the chimeras: W18N-W25C and W25N-W18C. These transformed leaves were analyzed under a confocal laser scanning microscope: yellow fluorescence (YFP) indicates interaction (BiFC), and red fluorescence (RFP) serves as a transformation control. Boxplots represent the relative fluorescence ratio (%YFP/RFP). Sample size (n) is indicated in the figure and represents independent biological replicates. In both (**B**) and (**C**), one-way ANOVA followed by Tukey’s HSD post-hoc test was performed. Different lowercase letters denote statistically significant differences among groups (p ≤ 0.05).

Next, we wondered how the repressor and activator functions of WRKY18 and WRKY25 are achieved. WRKY25 belongs to the group I family members and contains two DNA-binding domains (DBDs) while WRKY18 belongs to group II and has only one DBD in the more C-terminal part of the protein. For the closely related group I WRKYs, in which all contain two DBDs domains, it was shown that both domains can bind to DNA^33,34^. However, it remains unclear whether these different domains have distinct activator or repressor functions in plants. Therefore, we employed a domain-swapping approach focusing on the structural domains of these WRKYs. We tested different deletion and chimeric constructs in the transactivation assay in Arabidopsis protoplasts for their impact on the *WRKY53* promoter driving the *GUS* expression. These constructs were created by deleting parts of the coding sequences of *WRKY25* or by exchanging the coding sequences for the N-terminal and C-terminal regions between *WRKY18* and *WRKY25* (Fig. 3A and S3A).

The deletion construct W25N*, which lacks both DBDs and the C-terminal region of WRKY25, was unable to activate the expression of *WRKY53*. A similar lack of activation was observed with W25ΔpD1, which lacks a large part of the N-terminal DBD1, and W25ΔD2, which lacks the C-terminal DBD2 of WRKY25 (Fig. S3B). Interestingly, the chimera W18N-W25C downregulated *WRKY53* expression, whereas W25N-W18C upregulated it, mimicking the activator effect of the native WRKY25 protein. (Fig. 3B). These results suggest that, although both DBDs appear to be necessary for WRKY25 function, the N-terminal domain plays a critical role in activating *WRKY53* expression.

In addition, the effect of the two chimeras on the expression of *WRKY18* and *WRKY25* were tested in the transactivation assay. The two chimeras were used as effector constructs in a transactivation assay with a dual luciferase reporter system in Arabidopsis leaf protoplasts. For these assays, constructs harboring *WRKY18* and *WRKY25* promoters driving the expression of firefly luciferase, respectively, were co-transfected with effector constructs of WRKY18, WRKY25, WRKY53, W18N-W25C, and W25N-W18C. Consistent with our previous observations using *GUS* as reporter gene (Fig. 1A and 2B), the dual luciferase assays showed similar results with the native WRKY18, WRKY25 and WRKY53 on the promoter of *WRKY25* and *WRKY18* (Fig. S4A, B). For W18N-W25C and W25N-W18C the expression of the reporter gene driven by the *WRKY18* promoter was slightly upregulated, whereas reporter gene expression driven by the *WRKY25* promoter was downregulated in both cases (Fig. S4A, B). This demonstrates for these two promoters, that both, C-terminal or N-terminal domains of WRKY25, can mimic the effect of the wildtype WRKY25 and can override the WRKY18 effect.

To assess the influence of different protein domains on protein–protein interactions, the deleted and the chimeric proteins were analyzed using the BiFC system in *N. benthamiana* leaves, as previously described. Protein–protein interactions were observed in all cases, except for WRKY25 with W25ΔpD1 and the chimera W18N-W25C. In the case of the deletion construct, only a weak interaction was detected compared to the strong intensity observed in WRKY18 homodimers or WRKY18/WRKY25 heterodimer, based on the YFP/RFP fluorescence intensity ratio calculated from microscopy images. Interestingly, the chimera W25N-W18C interacted with both WRKY18 and WRKY25, to a similar extent as the WRKY18 homodimers or WRKY18/WRKY25 heterodimer. In contrast, W18N-W25C did not show any interaction with WRKY25 (Fig. 3C and S5). The chimera W18N-25C, which carries the C-terminal domain of WRKY25, did not interact with either WRKY18 or WRKY25 (Fig. 3C and S5). The observed interaction between W25N-W18C and WRKY25 is particularly noteworthy, given that WRKY25 does not homodimerize (Fig. 1B, 3C and S1, S5), and W18N-W25C failed to interact with WRKY25. (Fig. 3C and S5). These results suggest that the N-terminal domain of WRKY25 plays a significant role in protein–protein interactions, while the C-terminal domain of WRKY25 may abolish an interaction. All these results reinforced that the N-terminal domain of WRKY25 is more important for the modulation of the subnetwork.

### The C-terminus of WRKY25 prevents an overshoot of WRKY53 expression while the N-terminus of WRKY25 modulates a fine-tuning in the senescence

In order to characterize the impact of this subnetwork on senescence in more detail, we complemented *wrky25* mutant plants with the deletion constructs (*wrky25*:W25N*, *wrky25*:W25ΔpD1, *wrky25*:W25ΔD2) and the two chimeric constructs (*wrky25*:W18N-W25C and *wrky25*:W25N-W18C), alongside the native WRKY25 (*wrky25*:W25) as control. All complementing constructs were driven by the *UBIQUITIN10* promotor. All lines were grown alongside the Col-0 wild type and the *wrky25* mutant for comparison and the senescence phenotype was assessed by evaluating leaf color, chlorophyll content and maximum photochemical quantum yield of photosystem II (PAM fluorometry) over development using 6 to 8 plants per line for these analyses.

First, we compared the complementation line carrying the native *WRKY25* construct (*wrky25*:W25) with the wild type plants Col-0 and the *wrky25* mutants. The 4-week-old plants showed similar phenotypes across all lines. However, after 6 to 8 weeks, the *wrky25*:W25 plants displayed a phenotype more similar to Col-0, than to the *wrky25*, which exhibited accelerated senescence. This trend was consistent in all measurements: i) the automated colorimetric assay (ACA), a tool developed by our group that automatically identifies and quantifies the different colors of each leaf within a rosette by measuring the number of pixels corresponding to each color category (green, green/yellow, yellow, brown/dry, and purple) ii) chlorophyll content and iii) photosystem II functionality using pulse amplitude modulation (PAM) fluorometry (Fig. S7A-C). Col-0 and *wrky25*:W25 generally did not show significant differences (Fig. S6A, S7A), indicating that the transformed *WRKY25* construct substituted for the loss of a functional WRKY25 protein in *wrky25* mutant plants.

**Fig. 4:**
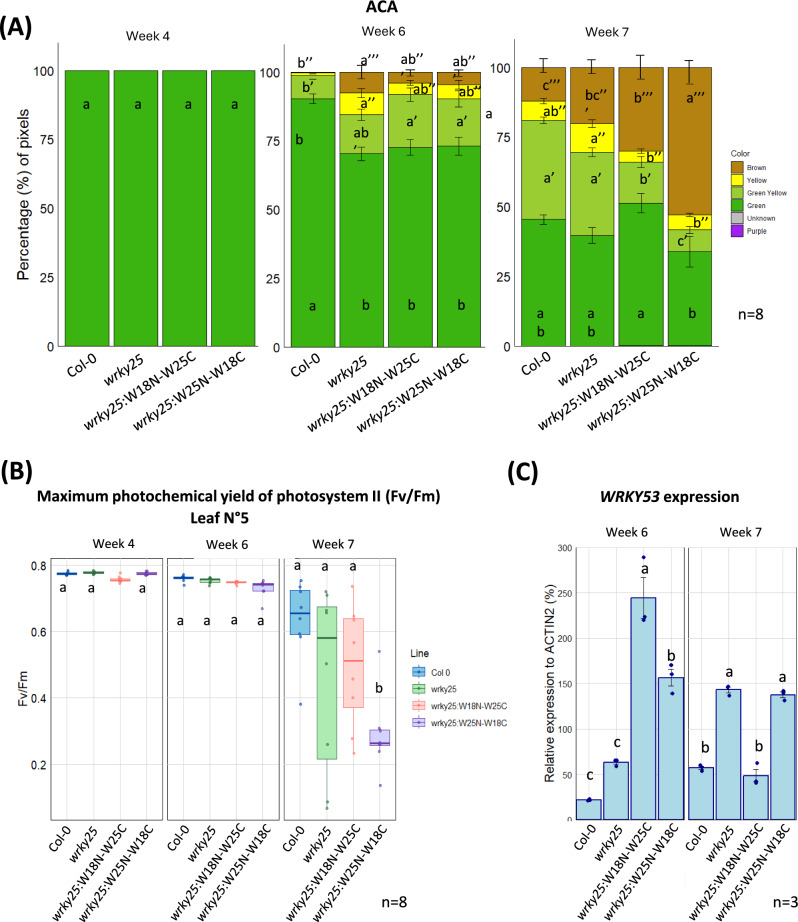
Senescence phenotyping of the complementation lines: *wrky25*:W18N-W25C and *wrky25*:W25N-W18C compared with Col 0 and *wrky25. (A)* The Automated Colorimetric Assay (ACA) categorizes the pixels corresponding to the color of individual leaves from 8 plants into five categories: green, green-yellow, yellow, brown, and purple. Quantification is presented as the percentage of each category relative to the total pixel number across all leaves (n = 8 biological replicates). **(B)** Boxplots present Fv/Fm values measured with PAM fluorometry for leaf No. 5 from 4-, 6-, and 7-week-old plants (n = 8 biological replicates). One-way ANOVA followed by Tukey’s HSD post hoc test was performed; different lowercase letters indicate statistically significant differences among groups (p ≤ 0.05). **(C)** Gene expression of *WRKY53* was analyzed by qRT-PCR and normalized to *ACTIN2* expression. Data are shown as mean ± SD (n = 3 biological replicates). Lowercase letters indicate statistically significant differences according to one-way ANOVA followed by Tukey’s HSD test (p ≤ 0.05).

Based on these results, we tested the complementation lines with the chimeric constructs (*wrky25*:W18N-W25C and *wrky25*:W25N-W18C) and also the deletion constructs (*wrky25*:W25N*, *wrky25*:W25ΔpD1, *wrky25*:W25ΔD2) alongside Col-0 and *wrky25*. Here, we used the entire rosette for the ACA. Initially, in 4-week-old plants, all lines exhibited uniformly green leaves (Fig. 4A, Fig. S7A). In 6-week-old plants, differences between the lines became apparent. In the *wrky25* mutant, the complementation lines *wrky25*:W25N*, *wrky25*:W25ΔpD1, *wrky25*:W25ΔD2, *wrky25*:W18N-W25C and *wrky25*:W25N-W18C, senescence was accelerated, as indicated by a reduced percentage of green leaves and an increased percentage of brown leaves compared to Col-0 (Fig. 4A, Fig. S6B, Fig. S8A), in which *wrky25*:W25ΔpD1 and both chimere complementation lines appear to be a bit less accelerated (Fig. 4A, Fig. S6B, Fig. S8A). The differences between the lines became even more pronounced in 7-week-old plants. While the *wrky25* mutant and the *wrky25*:W25N*, *wrky25*:W25ΔpD1, *wrky25*:W25ΔD2 lines continued to exhibit accelerated senescence, the *wrky25*:W18N-W25C line showed a deceleration in senescence progression, with a higher percentage of green leaves not only compared to *wrky25* but also to Col-0 (Fig. 4A, Fig. S6B, Fig. S8A). In contrast, the *wrky25*:W25N-W18C line displayed an even more accelerated senescence phenotype than *wrky25*, with a significantly higher percentage of brown leaves and a lower percentage of greenish leaves compared not only to Col-0 but also to *wrky25* (Fig. 4A).

In addition, the loss of photosystem II functionality was monitored over time. To ensure appropriate comparisons, leaves of defined positions within the rosette were selected for analysis. Leaf No. 5 (Fig. 4B, Fig. S8B, S9A) and No. 10 (Fig. S9B) were first used for PAM fluorometry, after which chlorophyll was extracted from these same leaves (Fig. S8C, S9B). Both parameters exhibited trends like those observed with the ACA. In leaf No. 5, the *wrky25*:W25N, *wrky25*:W25ΔpD1, and *wrky25*:W25ΔD2 lines showed a slightly faster decline in photosystem functionality compared to both Col-0 and the *wrky25* mutant (Fig. S8B). Although the differences were not statistically significant, the trend was consistent with the pattern observed in the ACA analysis. Additionally, the *wrky25*:W25N-W18C line exhibited a significantly faster decline in photosystem functionality compared to Col-0, and to a lesser extent, compared to the *wrky25* mutant. In contrast, the *wrky25*:W18N-W25C line behave more comparable to Col-0 (Fig. 4B). This accelerated loss of photosystem functionality in *wrky25*:W25N-W18C was also evident in leaf No. 10 at week 7, where it contrasted with both Col-0 and *wrky25* (Fig. S9A). A similar pattern was observed for the chlorophyll content (Fig. S8C, S9B). Again, no statistically significant differences were detected among the lines for this parameter, the *wrky25*:W25N-W18C line showed a slightly more pronounced decline in chlorophyll content by week 7 in leaves No. 5 and 10 compared to Col-0 and the *wrky25* mutant, whereas the *wrky25*:W18N-W25C line remained more comparable to Col-0 (Fig. S9B).

As the phenotyping parameters gave us more insights into influences of the different domains of WRKY25, the expression of *WRKY53* was monitored using qRT-PCR in Col-0, *wrky25*, *wrky25*:W18N-W25C, and *wrky25*:W25N-W18C at weeks 6 and 7. At week 6, *wrky25*:W18N-W25C exhibited the highest expression levels of *WRKY53* (Fig. 4C). This complementation line showed significantly higher levels of *WRKY53* compared to Col-0 and *wrky25*, and to a lesser extent, compared to the other complementation line, *wrky25*:W25N-W18C (Fig. 4C). Interestingly, by week 7, there was a shift in *WRKY53* expression, in which *wrky25*:W25N-W18C along with *wrky25* displayed the highest levels of *WRKY53*, surpassing not only Col-0 but also *wrky25*:W18N-W25C (Fig. 4C). It is also noteworthy that Col-0 and *wrky25* increased *WRKY53* expression from week 6 to week 7, while the opposite trend was observed in *wrky25*:W18N-W25C, which showed a reduction in *WRKY53* levels over the same period (Fig. 4C). However, this is consistent with the senescence phenotype, meaning that *WRKY53* mRNA increased and showed its peak expression earlier in the lines with accelerated senescence.

In conclusion, the senescence phenotypes revealed that only the W18N-W25C construct was able to rescue the phenotype of *wrky25* plants. In contrast, the deletion constructs exhibited senescence patterns similar to the *wrky25* mutant, while *wrky25*:W25N-W18C displayed an even more pronounced accelerated senescence phenotype. Together with the *WRKY53* expression data, these results suggest that both domains of WRKY25 are involved in senescence regulation, but in distinct ways: the N-terminal domain appears to be essential for activating *WRKY53* expression, whereas the C-terminal domain may serve to limit or fine-tune this activation to prevent excessive *WRKY53* expression.

### ROS molecules modulate the WRKY18/WRKY25/WRKY53 subnetwork

**Fig. 5:**
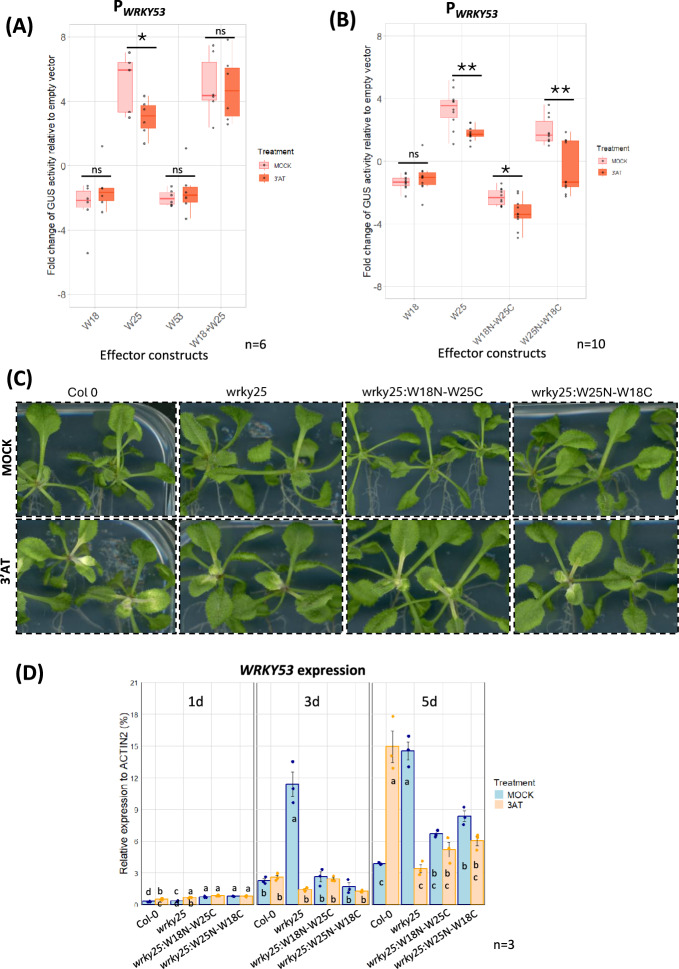
Oxidative stress effect on the transactivation assays in Arabidopsis protoplast for the P_*WRKY53*_ and the effect of oxidative stress *in planta* on young plants of the complementation lines *wrky25*:W18N-W25C and *wrky25*:W25N-W18C. Arabidopsis root protoplasts were transiently transformed with a fragment of the *WRKY53* (2759 bp) promoter fused to the *GUS* reporter gene, along with 35S:*WRKY18* (W18), 35S:*WRKY25* (W25), 35S:*WRKY53* (W53), or 35S:*WRKY18/WRKY25* (W18 + W25) **(A)** or, in a second series, 35S:*WRKY18* (W18), 35S:*WRKY25* (W25), 35S:*W18N-W25C* (W18N-W25C) and 35S:*W25N-W18C* (W25N-W18C) **(B)** as effector constructs. In both series, half of the transfected protoplasts were simultaneously incubated with 10 mM 3’-AT or the same volume of water for the MOCK conditions, respectively. The boxplots present the values relative to the empty vector control. Sample size (n) is indicated in the figure and represents independent biological replicates. Statistical significance was assessed using a two-tailed Student’s t-test (*p ≤ 0.05; **p ≤ 0.01; ns: not significant). **(C)** 2-week-old seedlings of *wrky25*:W18N-W25C and *wrky25*:W25N-W18C, Col-0 and *wrky25* were transferred onto new plates with or without 3’-AT. 5 days after transfer a bleaching of the leaves to a different extent in the different lines could be observed. **(D)** Gene expression of *WRKY53* with and without 3’-AT was analyzed by qRT-PCR in different lines and normalized to the expression of the *ACTIN2* gene (mean values ± SD, n = 3). A one-way ANOVA followed by Tukey’s HSD post-hoc test was performed. Different lowercase letters denote statistically significant differences among groups (p ≤ 0.05).

Whether a specific cue or signal is necessary for WRKY25 to exert its function within the subnetwork is still an open question. Previously, our group identified WRKY25 as a redox-sensitive protein. Moreover, it was demonstrated that *WRKY25* expression is induced by H₂O₂ treatment, whereas *WRKY25* overexpression at the same time reduces intracellular H_2_O_2_ contents^13^ indicating that WRKY25 is part of redox feed-back loop. Given its ability to sense oxidative signals, we hypothesize that WRKY25 might act as a switch within the subnetwork. To assess this in more detail, we conducted the previously used transactivation assay in Arabidopsis protoplasts under oxidative conditions. For this purpose, 3-Amino-1,2,4-triazole (3’-AT) was added after the transformation process to induce oxidative conditions. 3’-AT inhibits catalase activity, thereby increasing intracellular H₂O₂ levels within a physiological range, while having no effect on GUS activity measurement^13^. The upregulation of the reporter gene expression driven by the *WRKY53* promoter through the effector protein WRKY25 was significantly reduced under oxidative conditions. Surprisingly, the WRKY18/WRKY25 heterodimer did not significantly change its ability to upregulate *WRKY53* expression under oxidative conditions (Fig. 5A) even though the activity of the heterodimer was more similar to WRKY25 alone.

To decipher the influence of H₂O₂ on the activator effect of WRKY25, we first tested the influence of a cysteine at position 17 in the WRKY25 protein, which is the only cysteine not involved in the formation of the DNA-binding zinc finger structure. Therefore, this cysteine was replaced by a serine (W25cysmut) by site-directed mutagenesis using PCR. This change had no influence on the redox response of the WRKY25 protein indicating that this cysteine is most likely not involved in redox sensing (Fig. S10). We also tested the deletion constructs W25N*, W25ΔpD1, W25ΔD2, and the two chimeric constructs W18N-W25C and W25N-W18C in the same transactivation assay under oxidative conditions. Neither W25N* nor W25ΔD2 showed any difference in *WRKY53* expression (Fig. S11). W25ΔpD1 exhibited a slight but still negative effect but there is no statistically significant difference (Fig. S11). Intriguingly, in the case of the chimeras, we could observe that W18N-W25C consistently downregulated *WRKY53* expression, while W25N-W18C, which retains the N-terminal region and DBD1 of WRKY25, completely shifted its activity from a positive regulator under normal conditions to a negative regulator of *WRKY53* expression under oxidative stress (Fig. 5B).

The effect of H_2_O_2_ on the two chimeras was further investigated *in planta* using young plants to avoid the influence of other factors which are activated in older plants already undergoing senescence. Col-0, *wrky25*:W18N-W25C and *wrky25*:W25N-W18C seeds were grown on agar plates and seedlings were transferred to new media with and without 3’-AT after two weeks. Subsequently, they were photographed 1, 3, and 5 days later and harvested to evaluate the expression of *WRKY53* by qRT-PCR. After 5 days of treatment, degreening of the leaves was observed in Col-0 plants treated with 3’-AT in some of the leaves (Fig. 5C). However, in both complementation lines with the chimeric constructs only a milder or almost no effect of the oxidative conditions was observed (Fig. 5C). Especially, *wrky25*:W25N-W18C showed little to no leaf bleaching due to the oxidative stress (Fig. 5C). The expression of *WRKY53* increased over time in all 4 lines but this increase was much stronger in *wrky25*. After 5 days of 3’-AT treatment, *WRKY53* expression was highly induced in Col-0 plants compared to non-oxidizing conditions, while expression was even repressed by 3’-AT in *wrky25* mutant plants*.* This effect could already be observed after 3 days of 3’-AT treatment. The repressing effect was also visible after 5 days of 3’-AT treatment in *wrky25*:W18N-W25C and *wrky25*:W25N-W18C plants but to a lesser extend (Fig. 5B). Taken together, this suggests that both the N- and C-terminus of WRKY25 may be involved in the recognition of oxidative signals, in which the N-terminus appears to have a higher impact on activation of *WRKY53* expression and heterodimer formation.

### WRKY25 harbors putative redox switches in the N and C-terminal WRKY domain

Given that the WRKY25 protein can sense oxidative signals, we wondered which structural feature enables this function. It is well-documented that disulfide bonds formed by cysteine residues play a crucial role not only in the structural integrity and stability of proteins but also as redox switches that regulate protein function^35–37^. Interestingly, a novel redox switch involving a lysine-cysteine crosslink with a covalent NOS bridge has recently been identified as a potential regulatory element in proteins that modulate function in response to redox changes^35^. Furthermore, the NOS bridge was observed in proteins from diverse domains of life. In some cases, lysine residues were concurrently connected to two cysteine residues, resulting in the formation of a sulfur-oxygen–nitrogen-oxygen-sulfur (SONOS) bridge^38^.

**Fig. 6:**
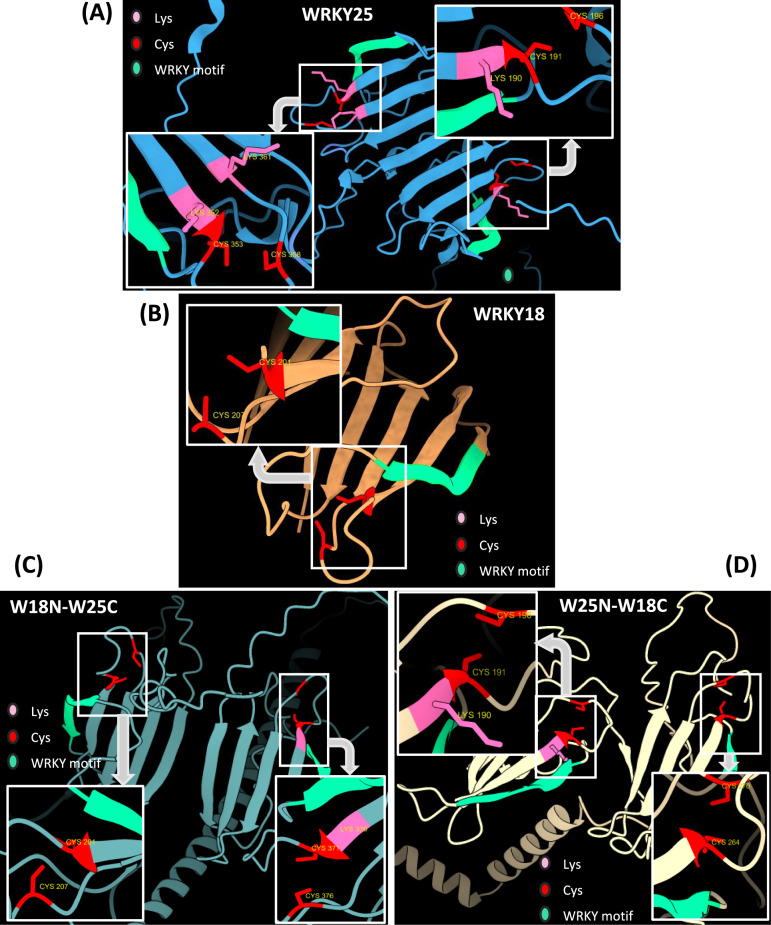
Alpha fold modeling of WRKY25, WRKY18 and the two chimeric proteins. **(A)** WRKY25 protein model illustrating the putative NOS bridge formed between Lys and Cys residues at the N-terminal region and at the C-terminal region. **(B)** WRKY18 protein model showing the presence of a Cys residue but the absence of the Lys residue is necessary to form the NOS bridge. **(C)** W18N-W25C chimeric protein model, depicting the putative NOS bridge in the C-terminal region. **(D)** W25N-W18C chimeric protein model, illustrating the putative NOS bridge in the N-terminal region. In all models, Lys residues are shown in pink, and Cys residues are shown in red, and the conserved WRKY motif (WRKYGQ) is depicted in mint. Grey arrows indicate closeups of the respective regions in the DBDs with the possible NOS bridges between Lys and Cys.

With this in mind, we modeled the WRKY25 protein using AlphaFold, along with two representative proteins (the rat Galectin-1 and the hunman hematopoietic cell receptor CD69) in which such redox switches had been previously identified^38^. Upon analyzing the predicted structures of Galectin-1 and CD69 and comparing them with the WRKY25 model, we identified a similar spatial arrangement of one lysine and two cysteine residues in WRKY25 (Fig. S12), suggesting analogous redox switches. Specifically, two potential lysine-cysteine redox switches were identified in WRKY25, each located within one of the WRKY domains at the N- and C-terminus of the protein (Fig. 6A).

Since WRKY18 appeared not to be redox-sensitive, we also modeled the WRKY18 protein for comparison. Notably, the predicted redox switch in the WRKY motif of WRKY25 was absent in WRKY18, which lacks the crucial lysine residue found in WRKY25 (Fig. 6B). We also modeled the two chimeras, and the putative redox switch was detected in the C-terminal of W18N-W25C (Fig. 6C) and in the N-terminal of W25N-W18C (Fig. 6D). Therefore, we speculate that this novel redox switch might be the key feature that allows WRKY25 to sense oxidative signals. In addition, we modeled the electrostatic surface potential of WRKY25 to assess whether the region or the amino acid residues involved in the potential redox switches exhibited reduced charge or a more neutral environment (Fig. S13), which could indirectly suggest the feasibility of bond formation. However, based on this analysis, we could not conclusively determine whether the formation of these redox switches is indeed possible.

Based on the evidence of these putative redox switches, targeted Liquid Chromatography-Mass Spectrometry (LC–MS) analysis was employed to verify their existence. Therefore, the N-8xHis-tagged recombinant WRKY25 protein was expressed in *E. coli* and purified. Subsequently, this recombinant protein was proteolytically cleaved under non-reducing conditions and the possible no-bridge and NOS-bridge peptides (Fig. S14A, B) were evaluated. If no bridge is formed, two separate peptides, SYFK and CTYPDCVSK, for DBD1 as well as SYYK and CTFQGCGVK for DBD2 should be detected. If a NOS bridge is formed, the two peptides would be linked resulting in a larger peptide, respectively. Mass/charge signals for all four no-bridge peptides of DBD1 and DBD2 could be detected (Fig. S14C) in the LC–MS chromatogram. In addition, peptide pairs for all possible four NOS-bridge combinations (DBD1: pos1 K-C1, pos1 K-C6, DBD2: pos2 K-C1 and pos2 K-C6) were also detected in the same LC–MS run (Fig. S14D). All represented transitions in Table S5 are unique for the four different NOS-bridge combinations except for the peptide/fragment pair m/z 524.89/588.25 (see method section for definition of uniqueness). The retention times of the K-C1 and K-C6 peptides were always similar as they are representing peptide isomers, which could not be separated on the LC–MS platform. Taken together, this is a strong indication that the NOS bridges are indeed formed.

## Discussion

Senescence is a very important developmental process at the end of plant development and is regulated in a very complex crosstalk to other processes. Besides almost all plant hormones, reactive oxygen species, especially H_2_O_2_, have been identified as important signaling components. A long-term increase in intracellular H_2_O_2_ contents has been characterized in Arabidopsis and rapeseed plants at the onset of senescence^6^ and genes responding to ROS are activated early in the chronology of gene expression changes^20^. However, how hydrogen peroxide signals are transmitted into transcriptional changes is a field with many open questions. Several transcription factors have already been characterized to change their DNA-binding affinity due to oxidative conditions, however, the detailed molecular mechanisms remain elusive in many cases.

A role for WRKY25 in senescence through the activation of *WRKY53* in a small subnetwork with the participation of WRKY18 has already been suggested, but the precise regulatory interactions among these WRKY transcription factors, including the role of WRKY18 as an effective repressor of *WRKY53*, had not been fully elucidated. In addition, previous studies have shown that *WRKY53* expression can be induced by H_2_O_2_ treatment and is upregulated in parallel to long-term increase in intracellular H_2_O_2_ contents at the onset of senescence^27,39^. Interestingly, WRKY25 has been characterized as a redox-sensitive transcription factor with a higher potential to activate *WRKY53* expression under more reducing conditions. Additionally, *WRKY25* expression is induced by H_2_O_2_, and *WRKY25* overexpression reduced intracellular H_2_O_2_ levels indicating complex regulatory feedback loops between H_2_O_2_ signals and WRKY transcription factors^13^. However, the precise mechanism by which WRKY25 senses and integrates oxidative signals within the WRKY18/WRKY25/WRKY53 subnetwork remained to be described.

Notably, WRKY25 belongs to the group I WRKY proteins, which are characterized by possessing two DBDs. We aimed to identify which domain was responsible for which regulatory function. Therefore, we created different deletion constructs and domain-swapping chimeras between WRKY18 and WRKY25. One chimera contained the N-terminal region of WRKY25 with its WRKY DBD1 (W25N-W18C), while the other chimera had the C-terminal region of WRKY25, which included its WRKY DBD2 (W18N-W25C). Our transactivation assays with the deletion constructs demonstrated that two DNA-binding domains are needed for a functional protein. The chimeric constructs revealed that the N-terminus of WRKY25 is necessary to activate *WRKY53* expression. Even though it was initially believed that only the C-terminal domains of group I WRKY proteins were responsible for DNA-binding^34,40^, subsequent research revealed that both WRKY DBDs (N- and C-terminal) are capable of binding DNA, with each displaying different binding specificities^33,41,42^. In our study, the chimera containing the N-terminus of WRKY25 retained this activating effect on *WRKY53* expression, similar to the native WRKY25, whereas the chimera with only the C-terminal DBD of WRKY25 lost this ability.

Another notable finding was WRKY25’s inability to homodimerize. However, WRKY25 did form heterodimers, particularly with WRKY18. Both chimeras were able to interact with native WRKY18, in which interactions involving the N-terminus of WRKY25 produced a stronger signal. In contrast, only W25N-W18C could form a dimer with WRKY25. This suggests a predominant role of the WRKY25 N-terminus in protein–protein interactions, while the C-terminus of WRKY25 appears to abolish the interactions with itself. This aligns with previous findings on the importance of N-terminal leucine zipper sequences in mediating WRKY-WRKY interactions^32,33,43^.

Another intriguing aspect was the activator effect of the WRKY18/WRKY25 heterodimer on *WRKY53* expression, despite WRKY18 alone or as homodimer acts as a strong repressor of *WRKY53* and its own expression. However, WRKYs are known to form homo- or hetero-complexes which exhibit varying DNA-binding activities and regulatory capabilities depending on the context^32^. For instance, WRKY18 enhances resistance to *Pseudomonas syringae*, but coexpression with WRKY40 or WRKY60 can increase susceptibility^44^. Moreover, it has been shown that WRKY60-WRKY18 interaction increases DNA-binding ability of WRKY18 while WRKY60-WRKY40 interaction decreases DNA-binding ability of WRKY40^31^. In our case, we speculate that WRKY25, in response to a signal, shifts the equilibrium between positive and negative effects on *WRKY53* expression within the subnetwork by sequestering WRKY18 to heterodimers, thereby reducing its repressor effect and increasing *WRKY53* expression.

To provide further insights into how WRKY25 modulates the subnetwork and *WRKY53* expression *in planta*, complementation lines of *wrky25* mutant plants were created using WRKY25, the deletion constructs and the chimeras between WRKY25 and WRKY18. As described before, the senescence phenotype of *wrky25* conflicts with its role as direct *WRKY53* activator, which clearly indicates that WRKY25 is part of a more complex regulatory network, in which the loss of a functional WRKY25 most likely leads to an imbalance in this network, making such a contradictory senescence phenotype possible^13^. However, for the analysis of the potential of various construct to complement the loss of a functional WRKY25 protein, this is not relevant. While the transformation of the wild type *WRKY25* construct could fully complement for the loss of a functional WRKY25 protein in the *wrky25* mutant, the accelerated senescence phenotype was not complemented by the deletion constructs and only partially by the two chimeric constructs. In 7-week-old plants, senescence was altered by transformation of both chimeras, respectively, but with different outcomes: while the presence of the N-terminus of WRKY25 even pronounced the accelerated senescence phenotype, the presence of C-terminus of WRKY25 started to delay senescence compared with *wrky25* or even Col-0. Consistently, *WRKY53* expression levels changed compared to Col-0 in different ways. These observations suggest that the two domains appear to have contradictory functions: the N-terminus of WRKY25 activates *WRKY53* expression while the C-terminus appears to prevent excessive *WRKY53* expression and fine-tunes the senescence process.

Further evidence from transactivation assays in Arabidopsis protoplasts revealed that the chimera containing the N-terminus of WRKY25 switched from an activator to a repressor of *WRKY53* expression under oxidative conditions. This was corroborated in young plants of the complementation lines which were exposed to oxidative stress through the inhibition of the catalases. Under these oxidative conditions, the Col-0 plants showed a very clear activation of the *WRKY53* expression compared to the two chimeras. While, under normal conditions, *WRKY53* expression in both chimeras was significantly higher than in Col-0, an additional activation of WRKY53 under oxidative conditions was not observed. This is consistent with the downregulation of *WRKY53* under oxidative conditions in the transactivation assays but also indicates that both, the C- and N-terminal domain of WRKY25 are involved in the transduction of the H_2_O_2_ signal and further supports that WRKY25 is most likely the driving force to balance of *WRKY53* expression and prevent overshooting reactions under oxidative stress conditions. Thereby, WRKY25 might also prevent a too early activation of premature senescence under stress conditions still contributing to the onset and progression of natural senescence.

Overall, our findings suggest that WRKY25 senses oxidative signals, specifically H₂O₂, through a structural feature, most likely a putative redox switch, which modulates the regulatory function, particularly influencing *WRKY53* expression. It is widely known that reactive oxygen species (ROS) can oxidize cysteine residues, which do not only play a role in scavenging ROS but also contribute to cellular signaling across various biological and pathological contexts^36^. Cysteine residues can form disulfide bonds, which are crucial for maintaining protein structure and stability, and can also function as redox switches that regulate protein activity^35–38^. Until recently, regulatory switches involving covalent crosslinks other than disulfide bridges had not been described. In this context, we tested a mutated version of WRKY25 in its cysteine at position 17 to evaluate the response under oxidative conditions. In the transactivation assays no change in the response to the oxidative conditions could be detected, indication that this cysteine appears to have no influence on the redox responsivity of WRKY25.

However, a novel covalent crosslink between a cysteine and one or two lysine residues, involving NOS or SONOS bridges, was recently identified as an allosteric redox switch^35,38^. The widespread occurrence of covalent lysine-cysteine redox switches has been identified in many proteins ranging from human to plant pathogenic proteins. For plants, a NOS bridge was suggested in the inositol monophosphatase from *Medicago truncatula*^38^ but this NOS bridge was not confirmed experimentally. NOS bridges have also been predicted in many different DNA-binding proteins, in which the formation of the NOS bridge mostly interferes with DNA–protein interaction^38^. Therefore, we tried to explore the potential presence of these novel redox switches in the WRKY25 protein, given its characterized sensitivity to oxidative signals. Using AlphaFold modeling, we identified two putative redox switches in WRKY25, located in the N-terminal and C-terminal DBDs. WRKY18, known as a non-redox-sensitive WRKY, lacked these putative redox switches, in fact, a lysine in the same position as the one found in the putative redox switches in the WRKY domain of WRKY25 is missing. As expected, the chimera W25N-W18C contained a redox switch at the N-terminus of WRKY25, consistent with the sensitivity to oxidative signals in the transactivation assays. Also, the chimera with the C-terminus of WRKY25 presented the putative redox switch which is consistent with its role *in planta* in the complementation lines. As an initial step to validate the existence of this redox switch, we digested purified recombinant WRKY25 proteins and analyzed the resulting peptides using LC–MS. Interestingly, we could support the presence of these putative redox switches, as the NOS-linked peptides could be detected. However, without isotopically labeled synthetic standard peptides, no quantitative conclusions can be drawn regarding the relative concentrations of specific peptides in the protein digest, as they may exhibit different response factors. Nevertheless, the detection of mass/charge signals (each verified by 5 to 9 transitions representing distinct peptide/fragment ion pairs) allows us to speculate that the NOS bridges in the WRKY25 protein really exist, but the evidence that changes in redox conditions lead to a reversible establishment of these NOS bridges is still pending. Establishing oxidative or non-oxidative conditions throughout the whole LC–MS procedure remains a significant challenge for further experimental analysis. At the same time, this limitation opens new questions and opportunities for a deeper investigation in this area.

**Fig. 7:**
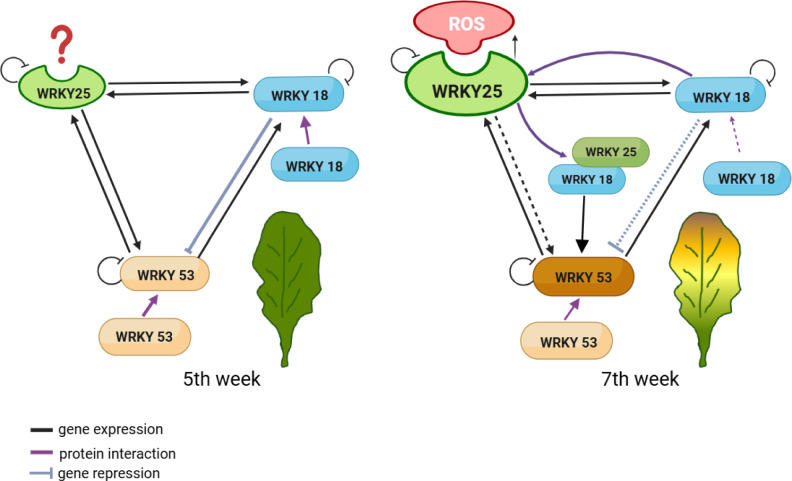
Model proposed for the subnetwork WRKY18/WRKY25/WRKY53 under the influence of an oxidative signal to induce senescence through WRKY53. The equilibrium of WRKY18/WRKY25/WRKY53 network encounters modifications at the onset of senescence. WRKY25 senses increasing H_2_O_2_ contents, and the oxidized form is less efficient in DNA-binding and upregulation of *WRKY53* expression. However, at the same time, increasing H_2_O_2_ contents lead to increasing *WRKY25* expression, so that the WRKY25/WRKY18 heterodimer forms, mitigating WRKY18’s repressive effect on *WRKY53* expression and increasing a positive effect on *WRKY53* expression. Additionally, WRKY25 and WRKY18 downregulate their own expression to prevent an excessive response. In this way, WRKY25 balances the expression and activity within the WRKY53/WRKY25/WRKY18 network to ensure a slow but progressive induction of senescence.

Based on the evidence presented, we propose a model (Fig. 7) for the interaction in a small subnetwork: WRKY25 senses oxidative stress and regulates *WRKY53* expression. Under oxidative conditions, WRKY25 is higher expressed but less efficient in activating *WRKY53* expression. However, as WRKY25 protein levels increase, the WRKY25/WRKY18 heterodimer forms, mitigating WRKY18’s repressive effect and further activating *WRKY53* expression. Additionally, WRKY25 and WRKY18 downregulate their own expression to prevent an excessive response. In this way, WRKY25 balances the expression and activity within the WRKY53/WRKY25/WRKY18 network to ensure a slow but progressive induction of senescence. Taken together, our results contribute to our mechanistic understanding of senescence regulation and suggest that the novel NOS redox switch also exists in plant regulatory proteins.

## Materials and methods

### Plant material and cultivation

Plants were grown on standard soil. An amount of 70 l of the standard soil CL Topf (Art.Nr.:10–00,300, PATZER ERDEN GmbH, Sinntal, Germany) was mixed with 8 l of sand (Flammer Bauunternehmung GmbH & Co. KG, Rheinsand, Tuebingen, Germany) and sieved with a mesh width of 8 × 10 mm. For all senescence phenotyping experiments the plants were grown under long-day conditions (16 h/8 h, light/dark), and moderate light intensity (80–100 μmol s⁻^1^ m⁻^2^) was applied in a climatic chamber at an ambient temperature of 20 °C. Individual leaf positions within the rosettes were color-coded with different threads, allowing for the analysis of individual leaves according to their age, even at very late stages of development^26,45^. The leaves were numbered starting from leaf No. 1 for the first true leaf, while the cotyledons were not considered in the enumeration. To avoid circadian effects, the plant material was always harvested at the same time of the day.

The lines used for senescence phenotyping experiments were: Col-0, *wrky25*, *wrky25*:UBI::W18N-W25C, *wrky25*:UBI::W25N-W18C, *wrky25*:UBI::W25. The Nottingham Arabidopsis Stock Centre (NASC) kindly provided seeds for Col-0 and the T-DNA insertion line of *WRKY25* (SAIL_529_B11; previously characterized in^46^) The complementation lines were produced for this manuscript as described below and seeds are available upon request.

For the experiments on the oxidative signal effect on seedlings, seeds of the different plant lines were sterilized first with 70% (v/v) ethanol, 0,05% (v/v) triton and subsequently with 100% ethanol. The sterilized seeds were grown on ½ Murashige and Skoog (MS) medium (1 l: 2.17 g MS micro and macro elements (Duchefa M0222.0025), pH 5.7–5.8, 8 g agar) with or without 20 µM of 3-Amino-1,2,4-triazole (3’-AT) added for oxidative conditions. For RNA extraction, two-week-old seedlings grown on MS medium without 3’AT were transferred to a new MS medium with or without 3’AT.

### Cloning and plant transformation

For ß-Glucuronidase reporter assays, a 3,000 bp promoter fragment upstream of the WRKY18 or WRKY25 start codon and a 2,759 bp sequence upstream of the WRKY53 start codon were cloned into the binary vector pBGWFS7.0 and used as reporter constructs, respectively. For the effector constructs, the cDNAs of WRKY18 (1701 bp, At4g31800), WRKY25 (1751 bp, At2g30250), WRKY53 (1501 bp, At4g23810) were cloned into the vector pJAN33. In addition, deletion constructs of WRKY25: W25N* (498 bp), W25ΔpD1 (1020 bp), W25ΔD2 (987 bp) and the chimeric constructs W18N-W25C (1239 bp) and W25N-W18C (1125 bp) were used also as effectors. Naturally occurring *Bsa*I restriction sites in *WRKY18* or *WRKY25* DNA sequences were mutated by site-directed mutagenesis. Deletion constructs of *WRKY25* were generated as follows. For W25N*, a fragment of 498 bp corresponding only to the N-terminal region up to (but not including) the first DNA-binding domain (DBD1) was amplified. For W25ΔpD1, two fragments were amplified: one from the N-terminal region upstream of DBD1 (429 bp), and one from the C-terminal region downstream of DBD1, containing the second DNA-binding domain (DBD2) (591 bp). For W25ΔD2, a fragment containing DBD1 from the N-terminal region just upstream of the DBD2 (939 bp), and a fragment from the C-terminal region downstream of DBD2 (48 bp), were amplified. For the chimeric constructs, the N-terminal (732 bp) and C-terminal (438 bp) sequences of *WRKY18*, and the N-terminal (681 bp) and C-terminal (489 bp) DNA sequences of *WRKY25*, were individually amplified. The respective fragments were ligated to generate the desired deletion and chimeric constructs. Additionally, a mutated version of *WRKY25* was generated in which the cysteine at position 17 was replaced with a serine via a site-directed G-to-C substitution using PCR.

All assembled fragments and the *WRKY25* version mutated at the Cys at pos. 17 (W25cysmut) were cloned into the pENTR-BsaI entry vector (described in^47^) using Golden Gate cloning. The deletion and chimeric constructs were subsequently transferred to the pJan33 destination vector via Gateway cloning. Correct insertions and ligation were verified by DNA sequencing. All primers used in these cloning steps are listed in Table S1.

For Dual Luciferase reporter assays, the same fragments upstream of the start codons of *WRKY18*, *WRKY 25* or *WRKY53* mentioned above, were cloned into the gateway destination vector pGWL7. The cDNAs of *WRKY18*, *WRKY25* and *WRKY53* were cloned into the gateway destination vector p2GW7 and used as effector constructs. In addition, the chimeric sequences generated for GUS reporter assays were used as template, amplified and cloned into the gateway vector p2GW7 to use the same vector for the effector constructs. All primers used for this cloning process are described in Table S1.

For Bimolecular Fluorescence Complementation (BiFC), the cDNAs of *WRKY18*, *WRKY25* and *WRKY53* as well as the deletion and chimeric sequences were cloned into the gateway vector pDONR221. Subsequently, they were cloned with the possible combinations of each other into the pBiFCt2in1-NN vector^48,49^ carrying both genes of interest. By this cloning step, the genes of interest were fused to the sequences encoding the N- or the C-terminal part of the yellow fluorescent protein (YFP), respectively. In the same vector backbone, an internal red fluorescent protein (RFP) gene is present as transformation and expression control. The expression of the fusion proteins is driven by the cauliflower mosaic virus 35S promoter. All primers used are presented in Table S2.

For plant complementation lines, the protein coding sequence of *WRKY25* (with previously removed *Bsa*I restriction site), the deletion, and chimeric sequences were cloned into the Green Gate entry vector pG00C (Table S3). The moderate constitutively active promoter of the *UBIQUITIN10* gene of Arabidopsis was used for all constructs as the 35S promoter led to gene silencing when combined with WRKY25^13^. All constructs were assembled into the final vector pZ03 with modular Green Gate technology described in^50^. The final vectors obtained were transformed into *Agrobacterium tumefaciens* strain GV3101. Finally, the transformed Agrobacterium was used to transform the *wrky25* mutant plants was transformed through floral dipping.

### Protoplast preparation and transformation

For the ß-Glucuronidase (GUS) reporter assays, the protoplasts were obtained from a root cell culture of *Arabidopsis thaliana* ecotype Col-0 as described before^49^. Protoplasts were transiently transformed using 20–40% (w/v) Polyethylenglycol (PEG1500) with different concentrations of the respective plasmid DNA as is described in^51^.

The protoplasts for the Dual-Luciferase reporter assay were obtained from fresh mesophyll tissue and transiently transformed with different concentrations of the respective plasmid DNA following the protocol published by^52^ with some minor variations in the transformation part. The number of resuspended protoplasts used for transformation was doubled as well as the amounts of PEG and W5 solutions. Finally, the protoplasts were resuspended in 250 µl instead of 1 ml of WI solution.

### ß-Glucuronidase reporter assay

Arabidopsis protoplasts from root cell culture were transformed using 5 μg of effector plasmid (pJAN33) and 5 μg of reporter plasmid (pBGWFS7) DNA. As an internal transformation control, 0.5 μg of a luciferase construct (pBT8-35SLUCm3) was co-transfected. After overnight incubation (15–17 h) in the dark at 20 °C, 10 ml of fall buffer (0.5 M mannitol, 15 m M MgCl₂, 5 mM MES) was added to the protoplasts, which were then collected by centrifugation (200 g, 4 °C). The collected protoplast pellet was subjected to lysis for protein extraction. 100 µl of lysis buffer (PROMEGA Luciferase Assay System E1500) was added to the pellet, followed by vigorous agitation using a vortex and incubation on ice for 5 min. The protein lysate was then concentrated by centrifugation (17,000 g, 10 min).

For luminescence measurements, 25 µl of Luciferase Assay Reagent (PROMEGA Luciferase Assay System E1500) was added to 20 µl of protein lysate. Luminescence was measured for 10 s using a TriStar2S Multimode Reader 941 plate reader (Berthold Technology). The fluorometric determination of β-Glucuronidase reporter activity followed the protocol described by^53^. The fluorometric measurements were performed using the same TriStar2S Multimode Reader 941 plate reader. To correct for transformation efficiency, β-Glucuronidase activity was normalized to luciferase luminescence. This was done by dividing the fluorometric measurements of each sample by their corresponding luminescence values. The resulting values were then normalized to those of an empty vector, which was used as a control. Additionally, GUS reporter assays with 3’-AT were performed as described above, except that 10 mM 3’-AT or an equivalent volume of water was added before the overnight incubation of the protoplasts.

### Dual-luciferase reporter assay

Arabidopsis protoplasts from mesophyll tissue were transformed using 4 µg each of effector (p2GW7), promoter (pGWL7), and internal transformation control (P2GW7-35S:RNLuc) plasmid DNA. After overnight incubation (15–17 h) in the dark at 20 °C, the protoplasts were collected by centrifugation at 200 g. For lysis, 100 µl of Passive Lysis Buffer (from Dual-Luciferase Reporter Assay System E1910 of PROMEGA) were used.

The measurements of both Firefly luciferase activity and Renilla luciferase activity were performed using a BertholdTech TriStar2S plate reader. Firefly luciferase activity was determined by adding 40 µl of Luciferase Assay Reagent II (LARII from Dual-Luciferase Reporter Assay System E1910) to the protoplast lysate and measuring the luminescence. Immediately afterwards, 40 µl of Stop & Glo® Reagent (SG from Dual-Luciferase Reporter Assay System E1910 of Promega) were added to the same sample, and the Renilla luciferase activity was measured.

To correct for transformation efficiency, the ratio of Firefly to Renilla luciferase activity was divided by the corresponding values of an empty vector (P2GW7-35S) used as a control. This step was performed to normalize the values. For reporter constructs, the same promoter fragments of the *WRKY18*, *WRKY25*, and *WRKY53* were used, but this time they were cloned into the vector p2GWL7.

### Transient transformation of Nicotiana benthamiana leaves

Suspension cultures of *Agrobacterium tumefaciens* containing the BiFC constructs were used to infiltrate *Nicotiana benthamiana* plants. Overnight cultures of *Agrobacterium tumefaciens* strain GV3101, which have been transformed with the BiFC constructs, were used to inoculate LB media containing the respective antibiotics. After 4–6 h of incubation, this culture was centrifuged at 18 000 g for 10 min. The bacterial pellet was diluted in infiltration media (10 mM MgCl2, 0.5 M MES, 100 mM Acetosyringone) to an OD_600_ of 0.5. Leaves of 4-week-old tobacco plants were infiltrated by manual injection using a 1-ml needleless syringe.

### Bimolecular fluorescence complementation (BiFC), confocal microscopy and cytometry

Assays with BiFC 2-in-1 constructs were used to study homo- and heteromeric interactions between the three WRKYs involved in our small subnetwork as well as with deletion W25N*, W25ΔpD1, W25ΔD2 and the domain swapping chimera between WRKY18 and WRKY25, W18N-W25C and W25N-W18C. In the same vector backbone, an internal RFP gene is present as transformation and expression control. Protein interactions were monitored using confocal microscopy. The interactions were detected and localized within the cells two days after the infiltration of the *N. benthamiana* leaves, as described above. At least three leaves of different plants were analyzed under the confocal microscope (LSM880, Zeiss, Jena, Germany) by using the preset sequential scan settings for YFP (Ex: 514 nm, Em: 517–553 nm) and for RFP (Ex: 561 nm, Em: 597–625 nm). The experiments were repeated at least three times. Images were acquired and analyzed using ZEN 3.0 (Blue edition) software (Zeiss). The mean fluorescence intensity of YFP and RFP was measured for each nucleus obtained from leaves of different plants. The YFP/RFP ratio was then calculated for each nucleus from independent biological samples. For each interaction type, at least four nuclei from distinct samples were analyzed.

Additionally, ratiometric BiFC assays were performed to ascertain the homodimeric interactions of the three WRKYs of our subnetwork mentioned above. The same pBiFCt-2in1-NN vectors^48,49^ carrying both genes of interest were used for transformation of Arabidopsis protoplasts. In this case, 8 µg of the plasmid DNA was used to express the fusion proteins. After overnight incubation in the dark, interactions were visualized by flow cytometry using CytoFLEX (Beckman Coulter, Brea, CA, USA). Both the internal mRFP and any reconstituted YFP were excited by the 488 nm laser. Peak emission was captured for YFP in FL1 (525/40 nm) and for RFP in FL3 (610/20 nm). All experiments were performed independently at least three times.

### Senescence phenotyping

To evaluate senescence phenotypes, various parameters indicating the state of senescence were considered. Leaves from seven to eight plants per time point were analyzed. The rosette leaves were detached and aligned according to their age, using a previously established color-coding system. These leaves were photographed, and an automated colorimetric assay (ACA) was used to group the pixels into four categories: green leaves (green), leaves starting to turn yellow (green-yellow), completely yellow leaves (yellow), and brown and/or dead leaves (brown/dead)^45^.

Leaves at positions 5 and 10 within the rosette were used to determine Fv/Fm values using the Imaging-Pulse-Amplitude-Modulation (PAM) method, indicating the activity of photosystem II (PSII) (PAM fluorometer Maxi version; ver. 2-46i, Walz GmbH, Effeltrich, Germany). These same leaves were also collected to determine the chlorophyll content as described in^45^.

### Gene expression analysis under oxidative conditions using qRT-PCR

Two-week-old seedlings grown on MS medium were transferred to a new MS medium with or without 3’-AT. These seedlings were collected after 1, 3 and 5 d, and total RNA was extracted with the GeneMATRIX Universal RNA Purification Kit (EURx) following the protocol provided by the manufacturer. Afterward, RevertAid RT Kit K1691 (Thermo Fisher Scientific Inc., Waltham, MA, USA) using oligo-dT primers was used for cDNA synthesis following the manufacturer’s instructions.

qRT-PCR was performed with the Master Mix KAPA SYBR® FAST following the manufacturer’s protocol in a thermal cycler CFX384 Bio-Rad (Bio-Rad Laboratories Inc., Hercules, CA, USA). The calculation method was the ΔΔCT described in^54^. In addition, the expression of the analyzed genes obtained was normalized to *ACTIN2* which has been characterized as a suitable reference gene for senescence^55^. The primers listed in Table S4 were used.

### Liquid chromatography-mass spectrometry (LC–MS)

The WRKY25 protein was ordered as N-terminally 8xHis-tagged proteins from Biomatik (Cambridge, Ontario, Canada). The protein was expressed in *E. coli* cells and purified by affinity purification (Biomatik, Canada). The quality and purification of the WRKY25 was controlled by SDS-PAGE, Coomassie staining and Western blotting followed by immune detection using anti-HIS antibodies^10^. This recombinant protein was used for the NOS-bridge peptide analysis. The authenticity of the protein was verified using non-targeted LC–MS profiling. All solvents used in the peptide profiling analyses were LC–MS grade.

Proteolytical protein digest: An in-solution digest of the recombinant protein (0.2 µg µl^−1^ in water) was performed by incubating 60 µl of the sample ON at 37 °C with 5 µl Trypsin (0.2 µg µl^−1^, proteomics grade, porcine; Merck) and 12 µl digestion buffer (7 µl 400 mM ABC buffer (NH4HCO3), 5 µl acetonitrile (ACN)). Finally, the protease digest was diluted 1:3 using 13% ACN in acidic water (1% (v/v) formic acid). As no Dithiothreitol (DTT) and iodoacetamide were added to the reaction, the digest took place under non-reducing conditions.

Targeted LC–MS Profiling Analysis: The targeted LC–MS profiling analysis was performed using a Micro-LC M5 (Trap and Elute) and a QTRAP6500 + (Sciex) operated in MRM (Multiple Reaction Monitoring) mode. Chromatographic separation was achieved on a HaloFused C18 column (150 × 0.5 mm (particle size 2.7 µm; 90 Å; Sciex) and a Luna C18(2) trap column (5 μm; 100 Å; 20 × 0.5 mm; Phenomenex) with a column temperature of 55 °C. The following binary gradient was applied for the main column at a flow rate of 16 µl min-1: 0—0.5 min, isocratic 98% A; 0.5—9 min, linear from 98% A to 60% A; 9—10 min, linear from 60% A to 5% A; 10—11 min, isocratic 5% A; 11—12 min, linear from 5% A to 98% A; 12—15 min, isocratic 98% A (A: water, 0.1% aq. formic acid; B: acetonitrile, 0.1% aq. formic acid). The samples were concentrated on the trap column using the following conditions: flow rate 25 µl min^−1^: 0—2.7 min isocratic 95% A; at 2.5 min start main gradient. The injection volume was 50 μl. Analytes were ionized using an Optiflow Turbo V ion source equipped with a SteadySpray T micro electrode in positive ion mode (ion spray voltage: 4800 V). Following additional instrument settings were applied: nebuliser and heater gas, nitrogen, 25 and 45 psi; curtain gas, nitrogen, 30 psi; collision gas, nitrogen, medium; source temperature, 200 °C; entrance potential, + 10 V; collision cell exit potential, + 10 V. The dwell time for all MRMs was 10 ms except for the trypsin autolysis control peaks, which were recorded with 5 ms. The declustering potential was kept at 80 V.

All MRM information was calculated using the Skyline 23.1 software^56^. Quantitative data extraction was performed using the vendor software Sciex OS. In the Skyline software all peptide transitions were screened for uniqueness against the protein sequences of the *E. coli* proteome, porcine trypsin and WRKY25 itself. All represented transitions in Fig S5 are unique for the four different NOS-bridge combinations except for the peptide/fragment pair m/z 524.89/588.25 which can be found on Pos1 K-C1 as well as in Pos1 K-C6. The transitions monitored for each peptide are shown in Table S5.

### Software used for modeling and statistical analysis of data

For Protein Modeling ColabFold was used following the indications of^57^. For viewing and manipulation of data obtained from ColabFold from proteins and chimeras PyMOL TM 2.5.5 (Schrödinger, LLC) was used. In addition, for modeling and determination of the electrostatic surface potential ChimeraX 1.9 was used. For the elaboration of constructs and sequence alignments CLC Main Workbench 21 (QIAGEN) was used. For analysis and image processing, ImageJ was used. In the case of ACA the leaves were individually processed in single images using a semi-automatic ImageJ macro described in^45^.

The statistical analysis was performed using EXCEL and R version 4.4.1 (The R Foundation for Statistical Computing). The specific statistical method was chosen based on the characteristics of the experiment. The statistical method used is detailed in the legends.

## Supplementary Information

Supplementary Information.

## Data Availability

All data generated or analyzed during this study are included in this published article (and its Supplementary Information files). DNA and protein sequence of all Arabidopsis WRKY factors are available at TAIR (https://www.arabidopsis.org/): WRKY25 (AT2G30250), WRKY18 (AT4G31800), WRKY53 (AT4G23810). Protein data on human hematopoietic cell receptor CD69 (PDB: 4GA9) and Galectin-1 of rats (PDB: 1E8I, chain A) are available at worldwide ProteinDataBank (https://www.wwpdb.org/).
