# Supplementary material for: The transcription factor WRKY25 can act as redox switch to drive the expression of WRKY53 during leaf senescence in Arabidopsis
Source: Sci Rep. 2025 Jul 29;15:27623. doi: 10.1038/s41598-025-13023-1 (PMC12307804; doi:10.1038/s41598-025-13023-1)
Supplement: Supplementary file 1 — Supplementary Information. [file 41598_2025_13023_MOESM1_ESM.pdf]

The transcription factor WRKY25 can act as redox switch to drive the expression of *WRKY53* during leaf senescence in Arabidopsis

Ana Gabriela Andrade Galan, Jasmin Doll, Edda von Roepenack-Lahaye, Natalie Faiss, and Ulrike Zentgraf *

Center for Plant Molecular Biology (ZMBP), University of Tübingen, Auf der Morgenstelle 32, 72076 Tübingen, Germany

***** Correspondence: ulrike.zentgraf@zmbp.uni-tuebingen.de

The following **Supporting Information** is available for this article:

Fig. S1: Homodimerization of WRK18, WRKY25, and WRKY53 using BiFC in transiently transformed Arabidopsis protoplasts and *Nicotiana benthamiana* leaves.

Fig. S2: Heterodimerization of WRK18, WRKY25, and WRKY53 using BiFC in transiently transformed *N. benthamiana* leaves.

Fig. S3. GUS transactivation assays in Arabidopsis protoplasts from root on the $P_{\textit{WRKY53}}$ and the effect of the deletions on it.

Fig. S4: Dual luciferase assays in Arabidopsis protoplasts from leaves on the $P_{\textit{WRKY18}}$ and $P_{\textit{WRKY25}}$ and the effect of the chimeras on them.

Fig. S5: Dimerization of WRK18 or WRKY25 with the deletion and chimeric versions, using BiFC in transiently transformed *N. benthamiana* leaves.

Fig. S6. Pictures of the leaves of representative plants of all lines used for senescence phenotype.

Fig. S7. Additional parameters used for senescence phenotyping of the complementation lines *wrky25:*W25 compared to Col-0 and *wrky25*.

Fig. S8. Additional parameters used for senescence phenotyping of the complementation lines *wrky25*:W25N*, *wrky25*:W25ΔpD1, *wrky25*:W25ΔD2 compared to Col-0 and *wrky25*.

Fig. S9. Additional parameters used for senescence phenotyping of the complementation lines *wrky25:*W18N-W25C and *wrky25:*W25N-W18C compared to Col-0 and *wrky25*.

Fig. S10. GUS transactivation assays in Arabidopsis protoplasts from root on the P_{WRKY53} and the effect of the Cys^{pos17} mutated version of WRKY25 under oxidative conditions.

Fig. S11. GUS transactivation assays in Arabidopsis protoplasts from root on the P_{WRKY53} and the effect on the deletions under oxidative conditions.

Fig. S12: Comparison of previously identified redox switches and the putative redox switches in WRKY25.

Fig. S13: Electrostatic surface potential map of WRKY25.

Fig. S14. Targeted LC-MS analysis of possible NOS-bridge peptides in WRKY25.

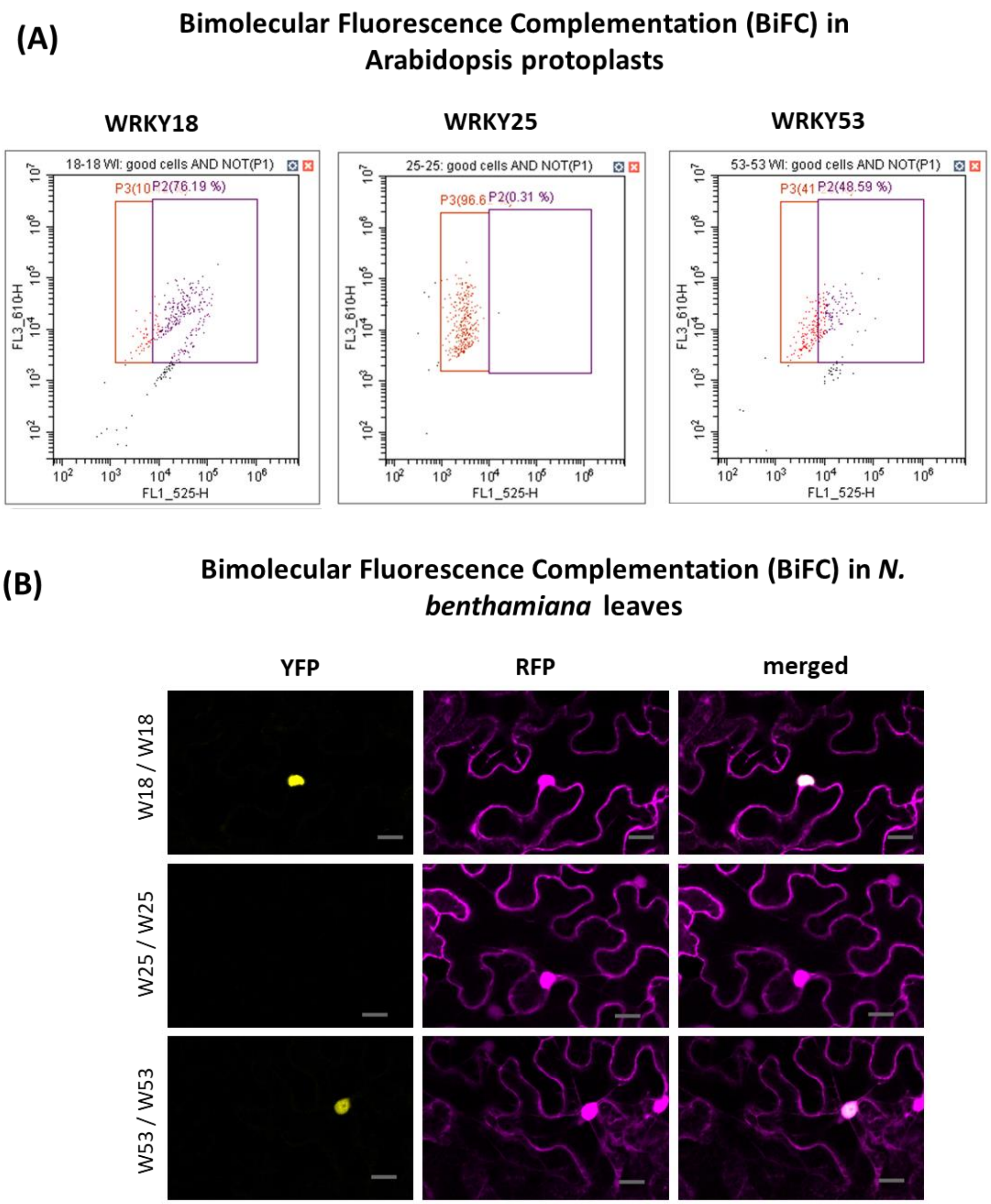

Fig. S1: Homodimerization of WRK18, WRKY25, and WRKY53 using BiFC in transiently transformed Arabidopsis protoplasts and *Nicotiana benthamiana* leaves.

(A) Arabidopsis protoplasts were transformed with pBiFCt2in1-NN constructs containing the possible combinations for the homodimerization of the three WRKYs of the subnetwork and were subsequently analyzed with the cytoflex cell sorter. The orange squares indicate transformed protoplasts (RFP), and purple squares indicate interaction via BiFC (YFP). **(B)** Leaves of *N. benthamiana* were transformed with pBiFCt2in1-NN constructs containing the possible combinations for the homodimerization of the three WRKYs of the subnetwork. These transformed leaves were analyzed under a confocal laser scanning microscope: yellow fluorescence (YFP) indicates interaction (BiFC), and red fluorescence (RFP) serves as a transformation control. Representative pictures are presented. The scale bar represents 20 µm.

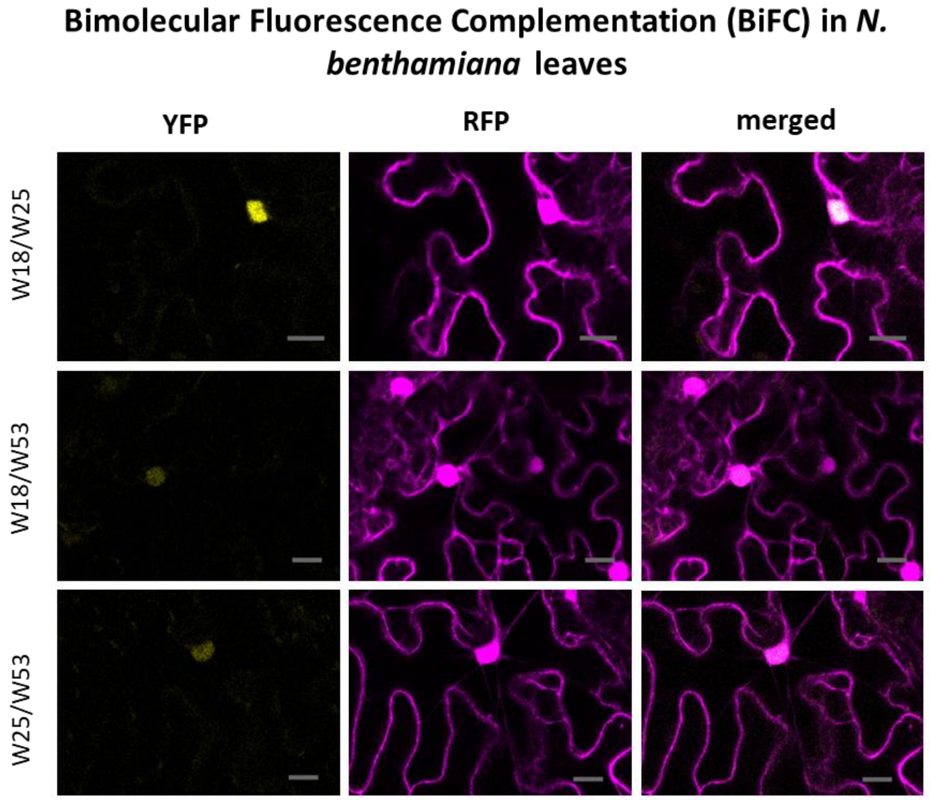

Fig. S2: Heterodimerization of WRK18, WRKY25, and WRKY53 using BiFC in transiently transformed *N. benthamiana* leaves.

Leaves of *N. benthamiana* were transformed with pBiFCt2in1-NN constructs containing the possible combinations for the heterodimerization of the three WRKYs of the subnetwork. These transformed leaves were analyzed under a confocal laser scanning microscope: yellow fluorescence (YFP) indicates interaction (BiFC), and red fluorescence (RFP) serves as a transformation control. Representative pictures are presented. The scale bar represents 20 µm.

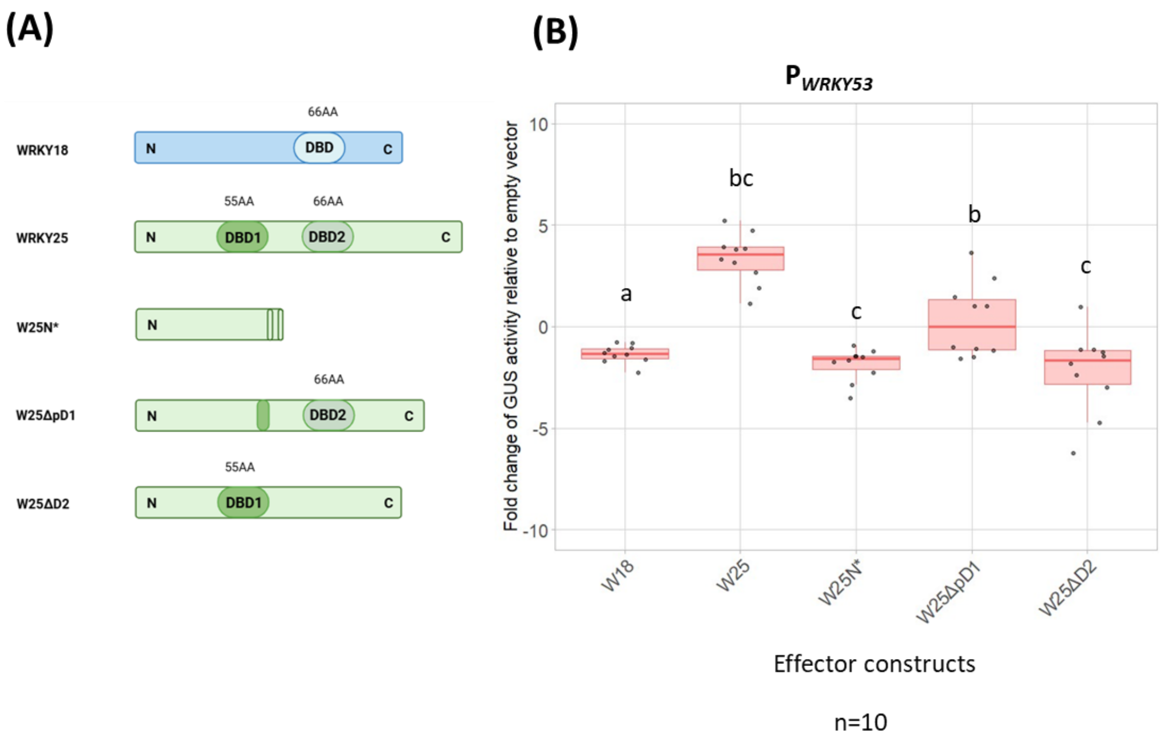

Fig. S3: GUS transactivation assays in Arabidopsis protoplasts from root on the P*$_{WRKY53}$* and the effect of the deletions on it.
(A) Schematic drawing represents the native WRKY18 and WRKY25 protein with their DNA-binding domains DBD1 and DBD2s well as deletions *W25N**, *W25ΔpD1,*and *W25ΔD2* **(B)** Arabidopsis protoplasts from root were transformed with a fragment of the promoter of *WRKY53* (2759 bp), fused to the *GUS* reporter gene, along with 35S:*WRKY18*, 35S:*WRKY25*, 35S:*W25N**, 35S:*W25ΔpD1,*or 35S:*W25ΔD2* as effector constructs. Values relative to the empty vector are presented as boxplots, with sample size (n) shown under the plot. The n represents independent biological replicates. One-way ANOVA followed by Tukey's HSD post-hoc test was performed. Lowercase letters indicate statistically significant differences between groups ($p \leq 0.05$).

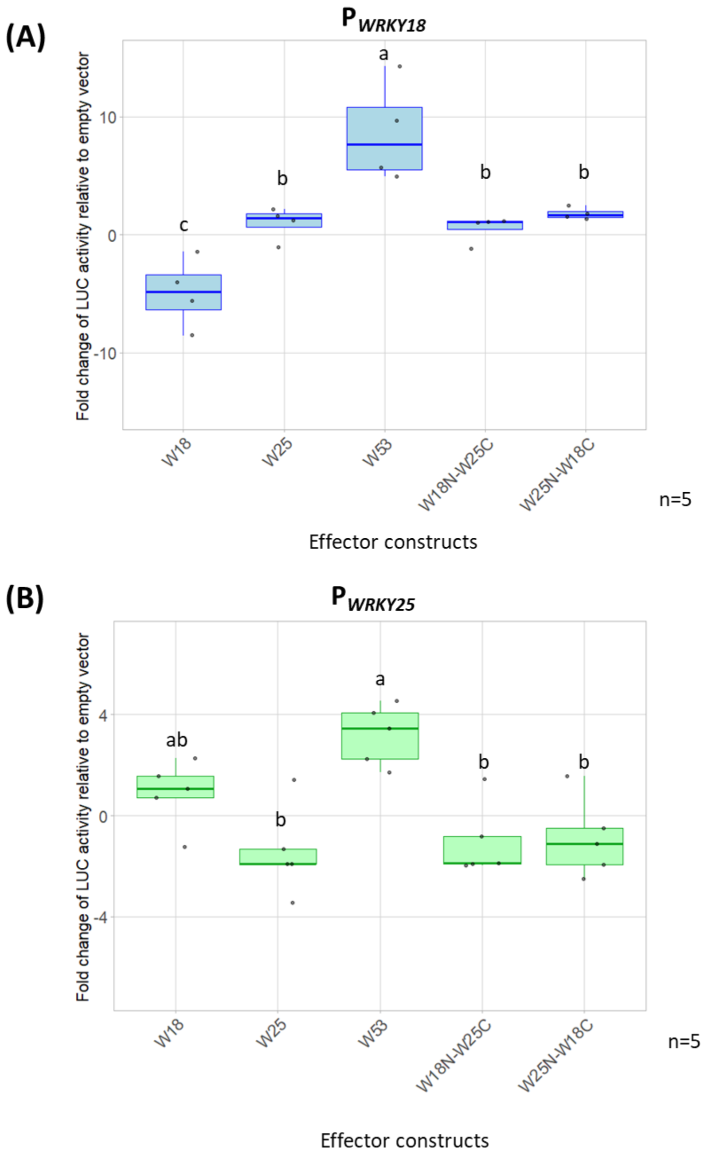

Fig. S4: Dual luciferase assays in Arabidopsis protoplasts from leaves on the P_{WRKY18} and P_{WRKY25} and the effect of the chimeras on them.
Arabidopsis leaf protoplasts were transformed with the fragment of the promoter of **(A)** *WRKY18* (3000 bp) or **(B)** *WRKY25* (3000 bp), fused to the firefly luciferase (*fLUC*) gene as a reporter construct, along with 35S:*WRKY18*, *35S:WRKY25*, 35S:*WRKY18*, 35S:*W18N-W25C* or 35S:*W25N-W18C* as effector constructs. Values relative to the empty vector are presented as boxplots, with sample size (n) shown at the right side of the plot. The n represents independent biological replicates. One-way ANOVA followed by Tukey's HSD post-hoc test was performed. Lowercase letters indicate statistically significant differences between groups ($p \leq 0.05$).

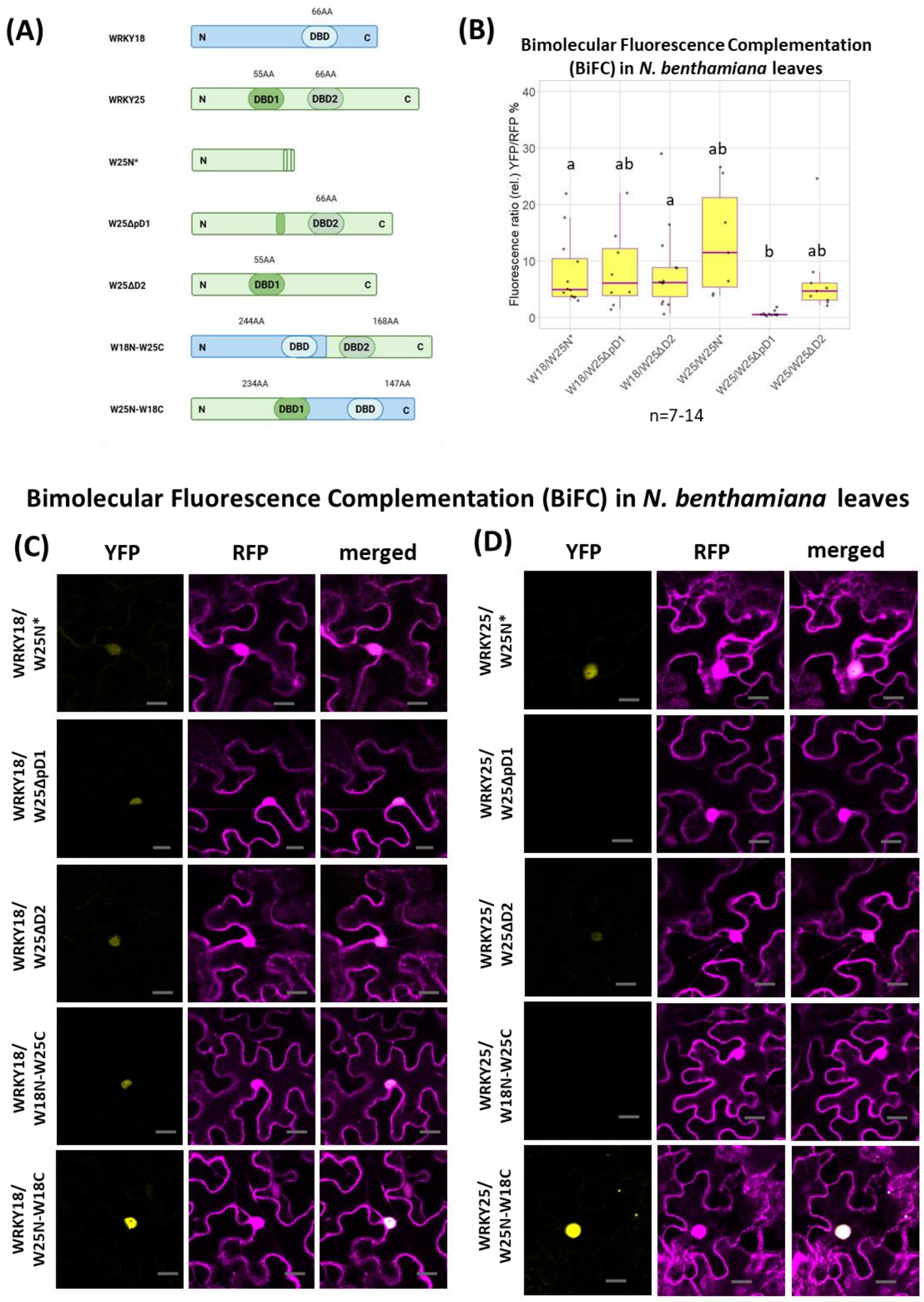

Fig. S5: Dimerization of WRK18 or WRKY25 with the deletion and chimeric versions, using BiFC in transiently transformed *N. benthamiana* leaves.

(A) Schematic drawing represents the native WRKY18 and WRKY25 protein with their DNA-binding domains DBD1 and DBD2s well as deletions *W25N**, *W25ΔpD1, W25ΔD2*, and the chimeras W18N-W25C and W25N-W18C. **(B)** Boxplots representing the relative fluorescence ratio (% RFP/YFP) are presented.

Sample size (n) is indicated under the plot and represents independent biological replicates. In both (B) and (C), one-way ANOVA followed by Bonferroni post hoc correction was performed. Different lowercase letters indicate statistically significant differences among groups ($p \leq 0.05$). **(C)** Leaves of *N. benthamiana* were transformed with pBiFCt2in1-NN constructs containing the possible combinations for the dimerization of WRKY18 with the deletions of WRKY25 and the chimeras of domain swapping between WRKY18 and WRKY25. These transformed leaves were analyzed under a confocal laser scanning microscope: yellow fluorescence (YFP) indicates interaction (BiFC), and red fluorescence (RFP) serves as a transformation control. Representative pictures are presented. The scale bar represents 20 µm. **(D)** Leaves of *N. benthamiana* were transformed with pBiFCt2in1-NN constructs containing the possible combinations for the dimerization of WRKY25 with the deletions of WRKY25 and the chimeras of domain swapping between WRKY18 and WRKY25. These transformed leaves were analyzed under a confocal laser scanning microscope: yellow fluorescence (YFP) indicates interaction (BiFC), and red fluorescence (RFP) serves as a transformation control. Representative pictures are presented. The scale bar represents 20 µm.

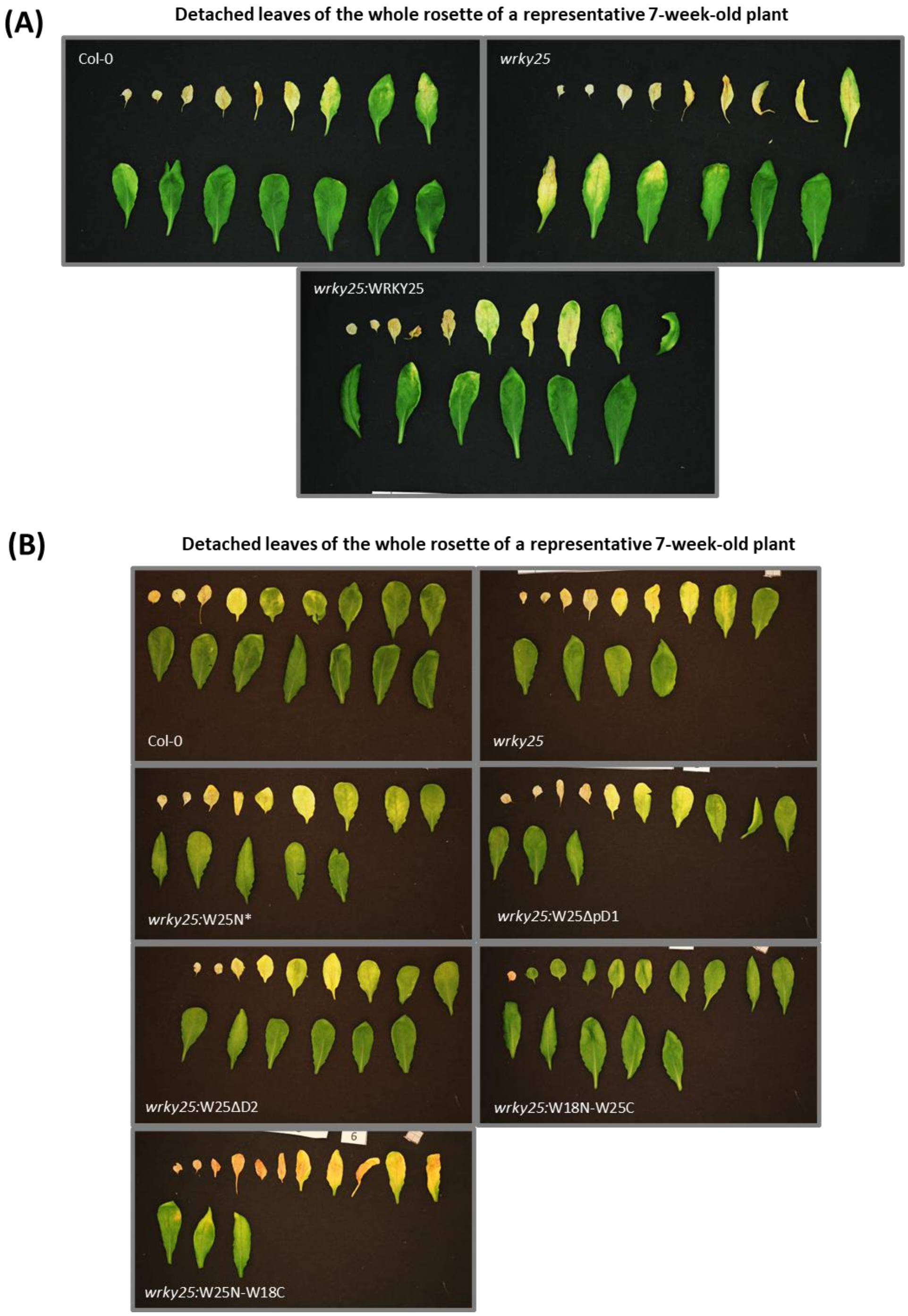

Fig. S6: Pictures of the leaves of representative plants of all lines used for senescence phenotyping.
(A) Representative pictures of all leaves of the whole rosette detached showing the differences between the complementation line: *wrky25:W25* compared with Col 0 and *wrky25*.
(B) Representative pictures of the whole rosette of the plants used for senescence phenotyping the complementation lines: *wrky25*:W25N*, *wrky25*:W25ΔpD1, *wrky25*:W25ΔD2, *wrky25*:W18N-W25C and *wrky25*:W25N-W18C compared with Col 0 and *wrky25*.

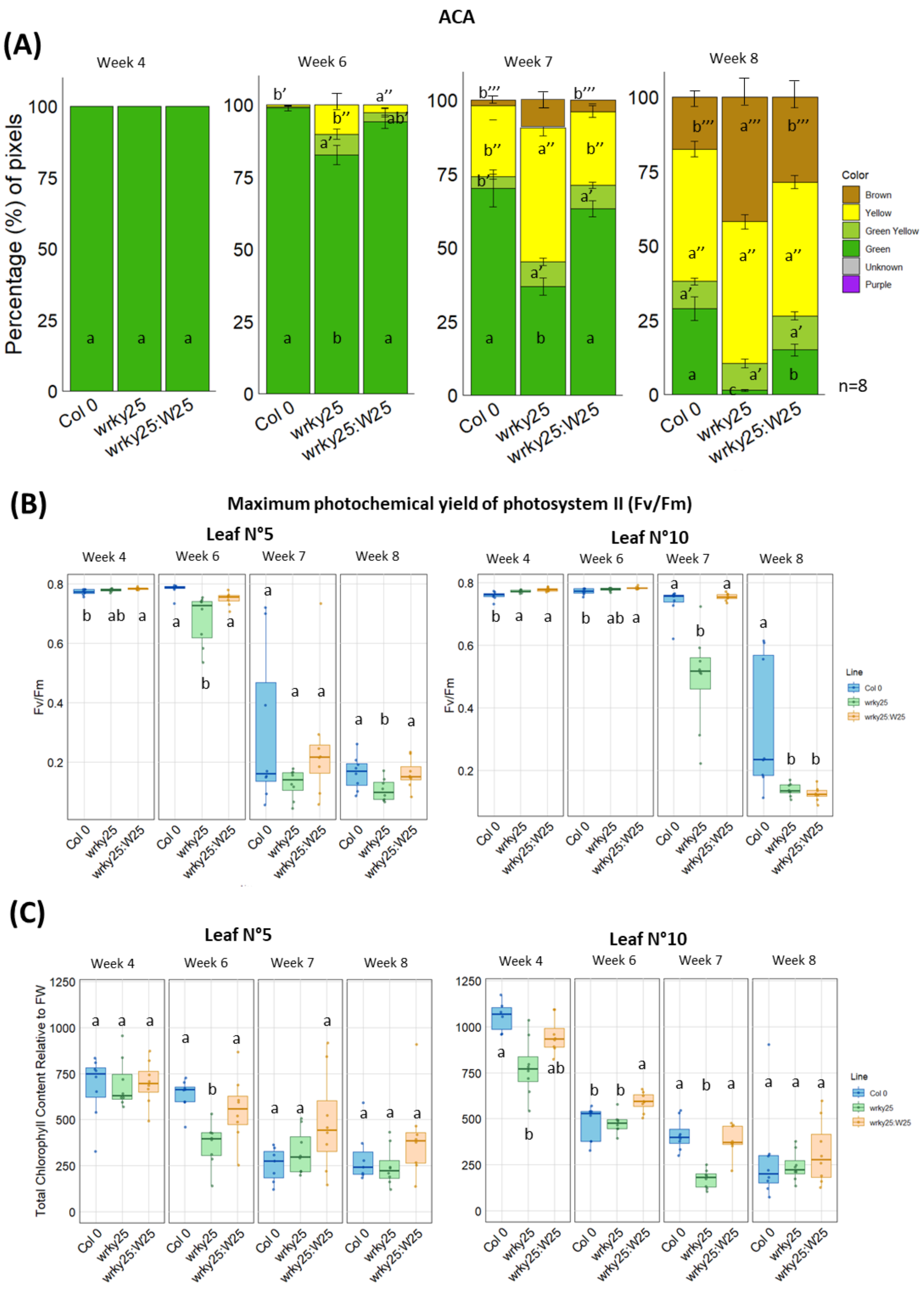

Fig. S7: Additional parameters used for senescence phenotyping of the complementation line *wrky25*:W25 compared to Col-0 and *wrky25*.

(A) The Automated Colorimetric Assay (ACA) categorizes the pixels corresponding to the color of individual leaves from 8 plants into five categories: green, green-yellow, yellow, brown, and

purple. Quantification is presented as the percentage of each category relative to the total pixel number across all leaves (n=8). **(B)** Boxplots of Fv/Fm values measured with PAM fluorometry for leaves No. 5 and No. 10 from 4-, 6-, 7-, and 8-week-old plants (n=8). **(C)** Chlorophyll content relative to fresh weight for leaves No. 5 and No. 10 from 4-, 6-, 7-, and 8-week-old plants (n=8). In all cases, n refers to independent biological replicates. Statistical analysis was performed using one-way ANOVA followed by Tukey's HSD post hoc test. Lowercase letters indicate statistically significant differences between groups ($p \leq 0.05$).

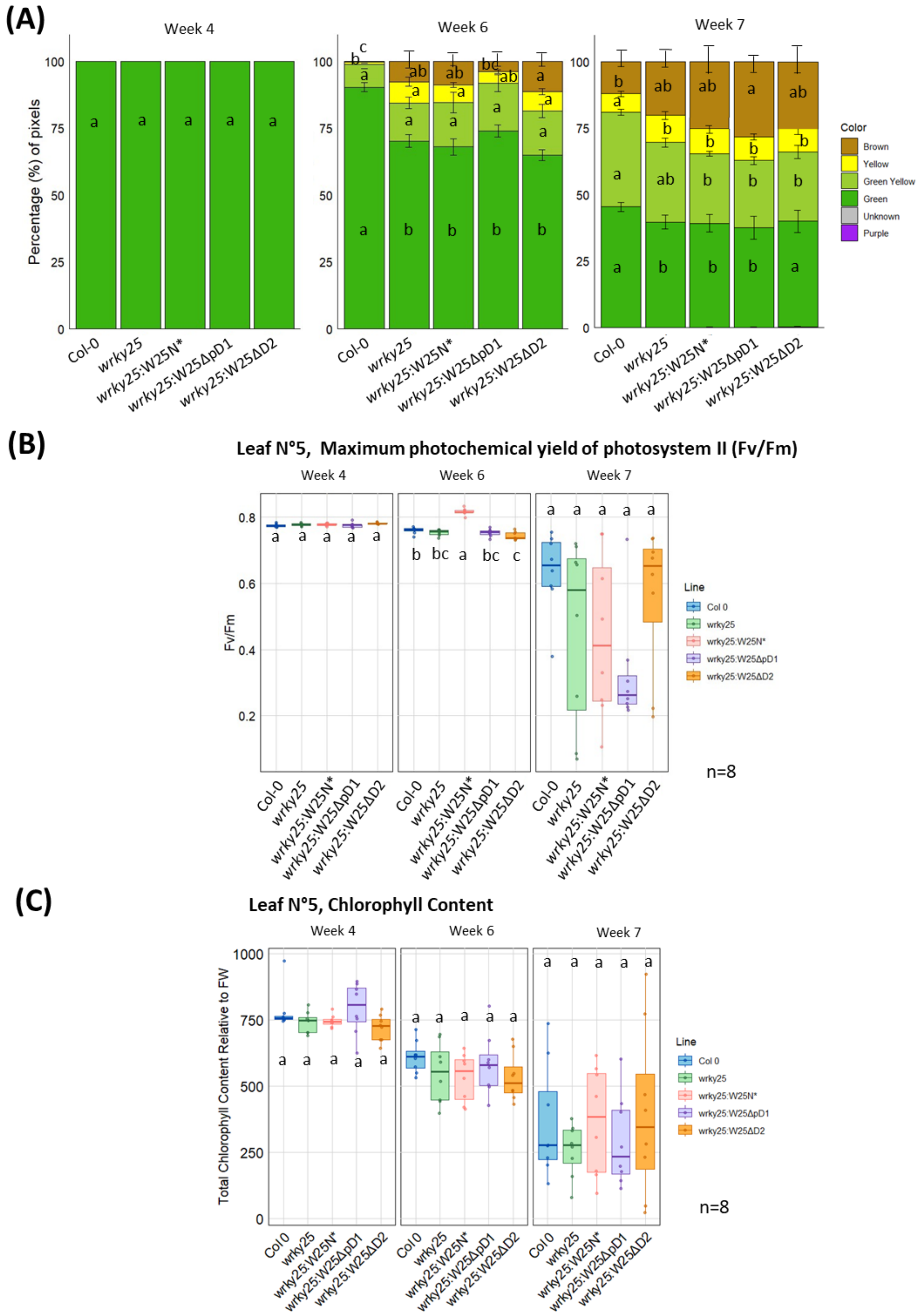

Fig. S8. Additional parameters used for senescence phenotyping of the complementation line *wrky25*:W25N*, *wrky25*:W25ΔpD1, *wrky25*:W25ΔD2 compared to Col-0 and *wrky25*. **(A)** The Automated Colorimetric Assay (ACA) categorizes the pixels corresponding to the color of individual leaves from 8 plants into five categories: green, green-yellow, yellow, brown, and

purple. Quantification is presented as the percentage of each category relative to the total pixel number across all leaves ($n = 8$). **(B)** Boxplots of Fv/Fm values measured with PAM fluorometry for leaves No. 5 from 4-, 6-, 7-, and 8-week-old plants ($n = 8$). **(C)** Chlorophyll content relative to fresh weight for leaves No. 5 from 4-, 6-, 7-, and 8-week-old plants ($n = 8$). In all cases, n refers to independent biological replicates. Statistical analysis was performed using one-way ANOVA followed by Tukey's HSD post hoc test. Lowercase letters indicate statistically significant differences between groups ($p \leq 0.05$).

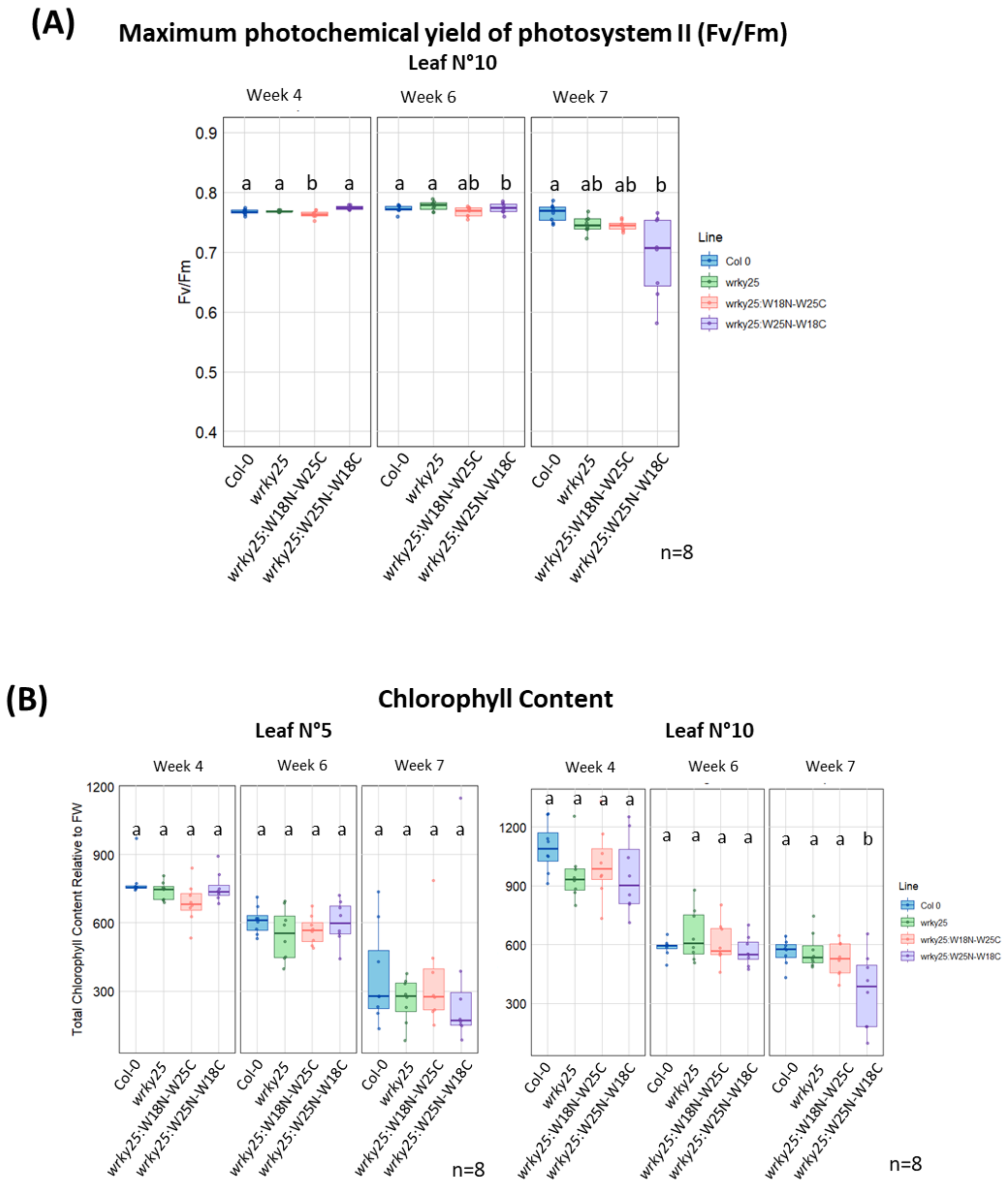

Fig. S9: Additional parameters used for senescence phenotyping of the complementation lines *wrky25*:W18N-W25C and *wrky25*:W25N-W18C compared to Col 0 and *wrky25*.
(A) Boxplots of Fv/Fm values measured with PAM fluorometry for leaf No. 10 from 4-, 6-, and 7-week-old plants (n=8). **(B)** Chlorophyll content relative to fresh weight for leaves No. 5 and No. 10 from 4-, 6-, and 7-week-old plants (n=8). In all cases, n refers to independent biological replicates. Statistical analysis was performed using one-way ANOVA followed by Tukey's HSD post hoc test. Lowercase letters indicate statistically significant differences between groups ($p \leq 0.05$).

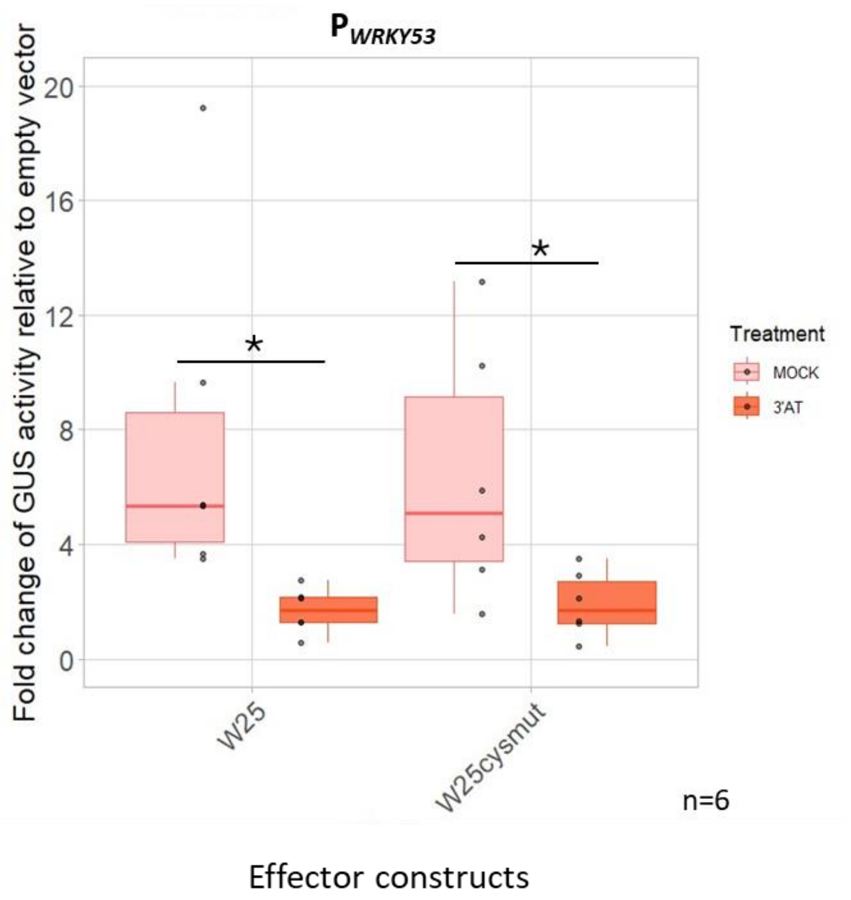

Fig. S10: GUS transactivation assays in Arabidopsis protoplasts from root on the P$_{WRKY53}$ and the effect of the Cys^{pos17} mutated version of WRKY25 under oxidative conditions. Arabidopsis protoplasts from root were transformed with a fragment of the promoter of *WRKY53* (2759 bp), fused to the *GUS* gene as a reporter construct, along with 35S:*WRKY25*, 35S:*W25cysmut*, as effector constructs. In this case half of the transfected protoplasts were simultaneously incubated with 10 mM 3'-AT or the same volume of water for the MOCK conditions, respectively. The values relative to the empty vector control are presented as boxplots (n=6), in which n refers to the number of biological replicates. Statistical significance was assessed using a two-tailed Student's t-test (*p ≤ 0.05; **p ≤ 0.01; ns: not significant).

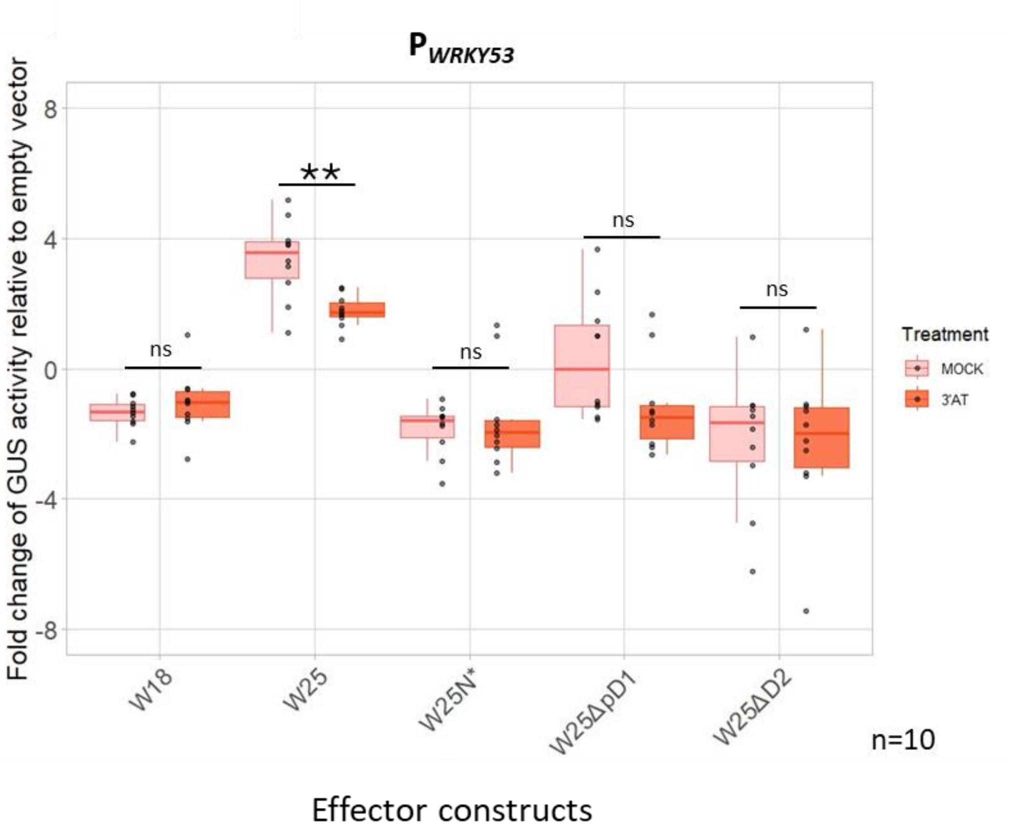

Fig. S11: GUS transactivation assays in Arabidopsis protoplasts from root on the P_{WRKY53} and the effect on the deletions under oxidative conditions.

Arabidopsis protoplasts from root were transformed with a fragment of the promoter of *WRKY53* (2759 bp), fused to the *GUS* gene as a reporter construct, along with 35S:*WRKY18*, 35S:*WRKY25*, 35S:*W25N**, 35S:W25ΔpD1, 35S:*W25ΔD2, as* effector constructs. In this case half of the transfected protoplasts were simultaneously incubated with 10 mM 3'-AT or the same volume of water for the MOCK conditions, respectively. The values relative to the empty vector control are presented as boxplots (n=10), in which n refers to the number of biological replicates. Statistical significance was assessed using a two-tailed Student's t-test (*$p \leq 0.05$; **$p \leq 0.01$; ns: not significant).

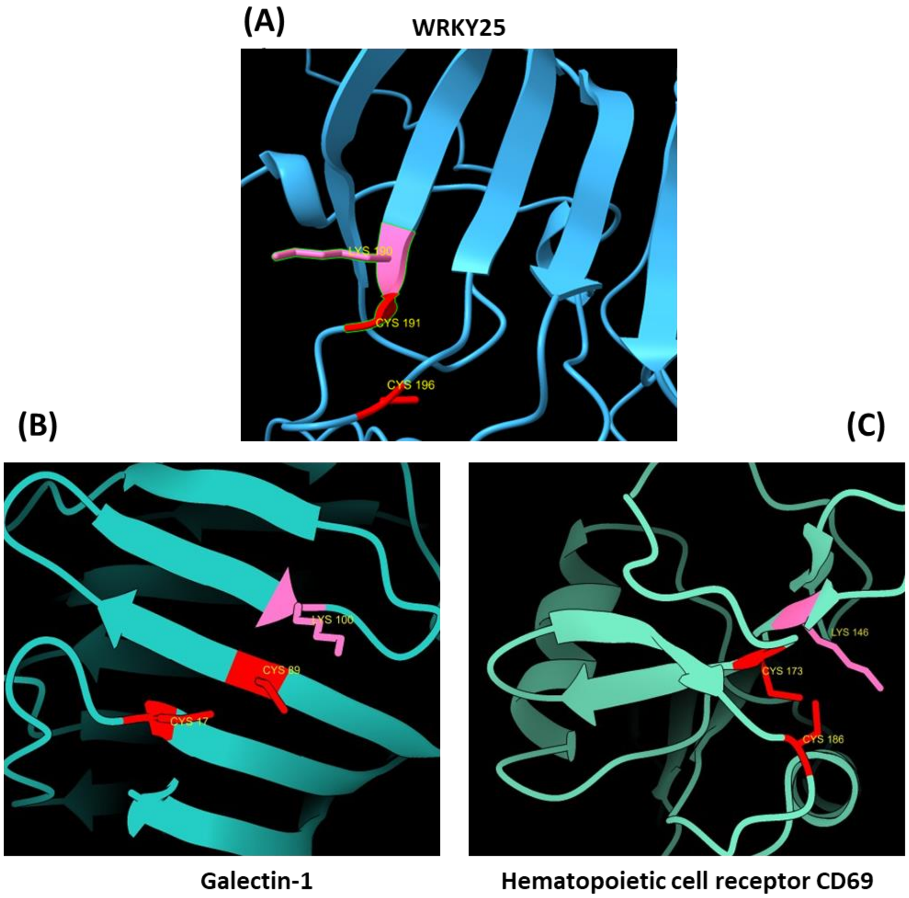

Fig. S12: Comparison of previously identified redox switches and the putative redox switches in WRKY25.

(A) Spatial arrangement of the potential redox switch located at the N-terminus of WRKY25. **(B)** Spatial arrangement of the redox switch previously identified in Galectin-1 of rats (PDB: 1E8I, chain A). **(C)** Spatial arrangement of the redox switch previously observed in the human hematopoietic cell receptor CD69 (PDB: 4GA9). In all models, LYS residues are shown in pink, and CYS residues are shown in red, and the conserved WRKY motif (WRKYGQ) is depicted in mint.

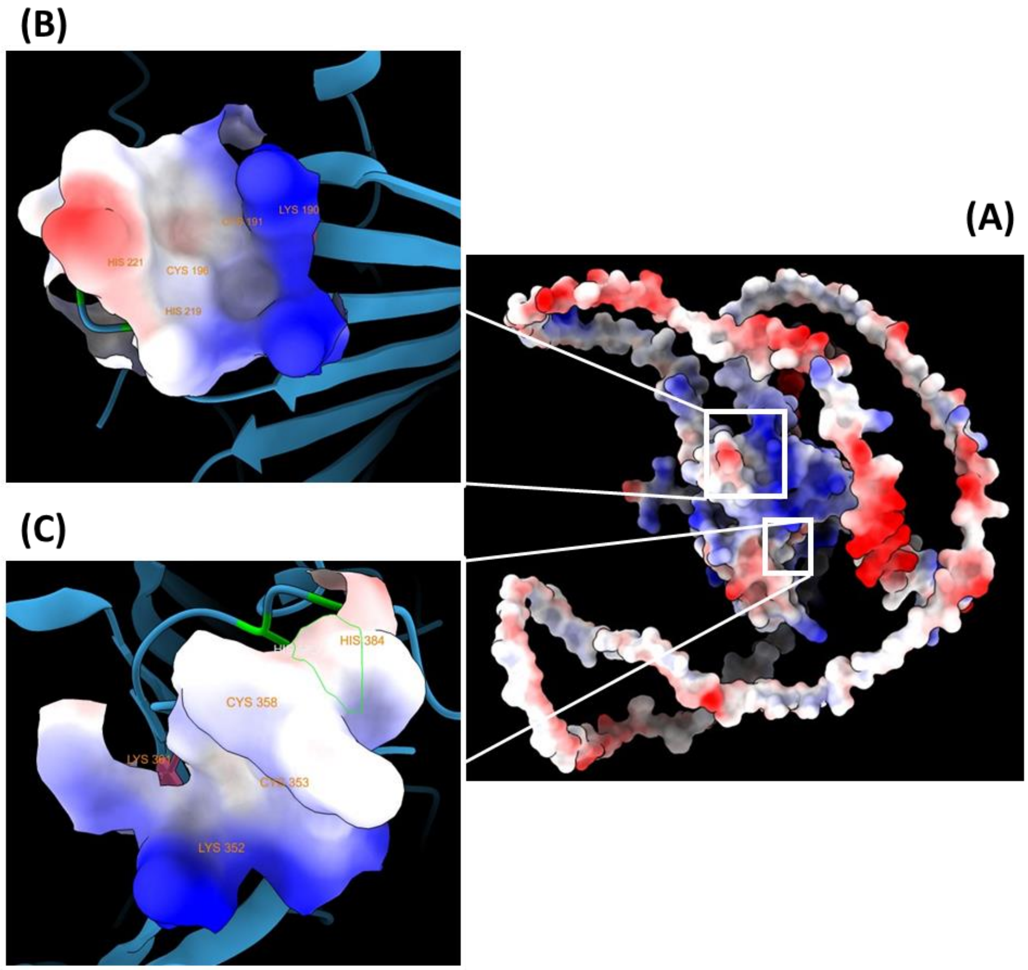

Fig. S13: Electrostatic surface potential map of WRKY25.
(A) Electrostatic surface potential of the full WRKY25 protein. **(B)** Electrostatic surface potential of the putative redox switch located at the N-terminus of WRKY25. **(C)** Electrostatic surface potential of the putative redox switch located at the C-terminus of WRKY25. Color scale: red indicates negatively charged regions, blue indicates positively charged regions, and white represents neutral or uncharged areas.

(A) WRKY25 amino acid sequence

MSSTSFTDLLGSSGVDCYEDDEDLRVSGSSFGGYYPERTGSGLPKFKTAQPPPLPISQSSHNFTFSDY
LDSPLLLSSSHSLISPTTGTFPLQGFNGTTNNHSDFPWQLQSQPSNASSALQETYGVQDHEKKQEMI
PNEIATQNNNQSFGTERQIKIPAYMVSRNSNDGYG**WRKY**GQKQVKKSENPRSYFK CTYPDCVSKKI
VETASDGQITEIIYKGGHNHPKPEFTKRPSQSSLPSSVNGRRLFNPASVVSEPHDQSENSSISFDYSDL
EQKSFKSEYGEIDEEEEQPEMKRMKREGEDEGMSIEVSKGVKEPRVVVQTISDIDVLIDGFR**WRKY**G
QKVVKGNTNPRSYYK CTFQGCGVKKQVERSAADERAVLTTYEGRHNHDIPTALRRS

(B) peptides after digestion

no-bridge pos1 peptides : SYFK (M+2H m/z 272.6); CTYPDCVSK (M+2H m/z 508.2)
no-bridge pos2 peptides : SYYK (M+2H m/z 280.6); CTFQGCGVK (M+2H m/z 471.7)
NOS-bridge pos1 K-C1 peptide: SYFK CTYPDCVSK (M+3H m/z 524.9)
NOS-bridge pos1 K-C6 peptide: SYFK CTYPDCVSK (M+3H m/z 524.9)
NOS-bridge pos2 K-C1 peptide: SYYK CTFQGCGVK (M+3H 505.9)
NOS-bridge pos2 K-C6 peptide: SYYK CTFQGCGVK (M+3H 505.9)

(C) LC-MS chromatograms for no-bridge peptides

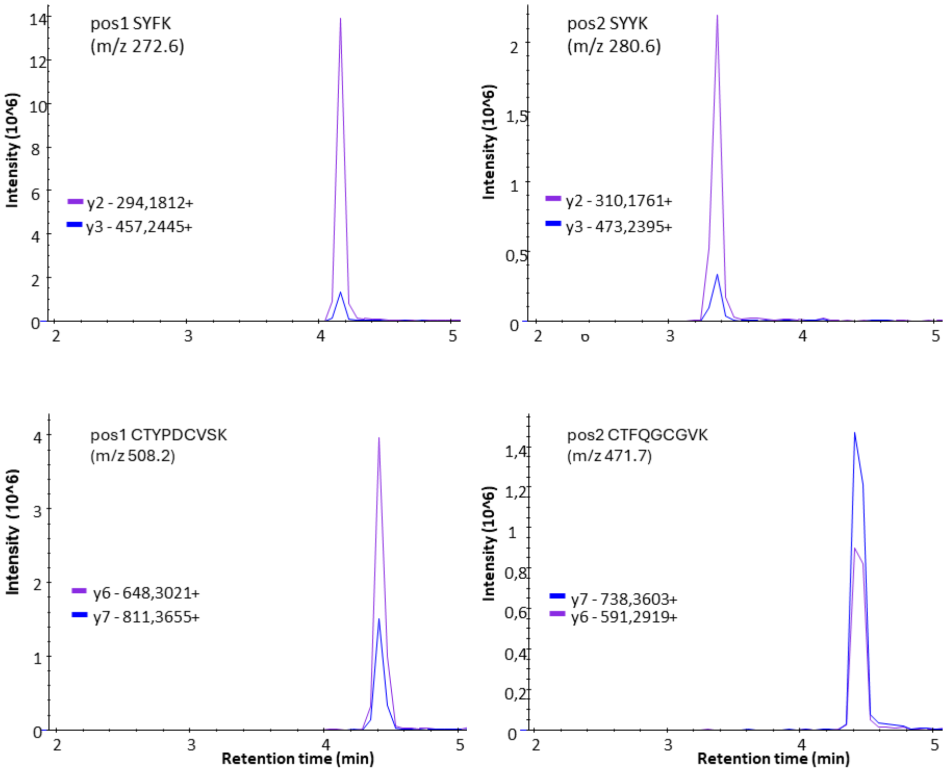

(D) **LC-MS chromatograms for NOS-bridge peptides**

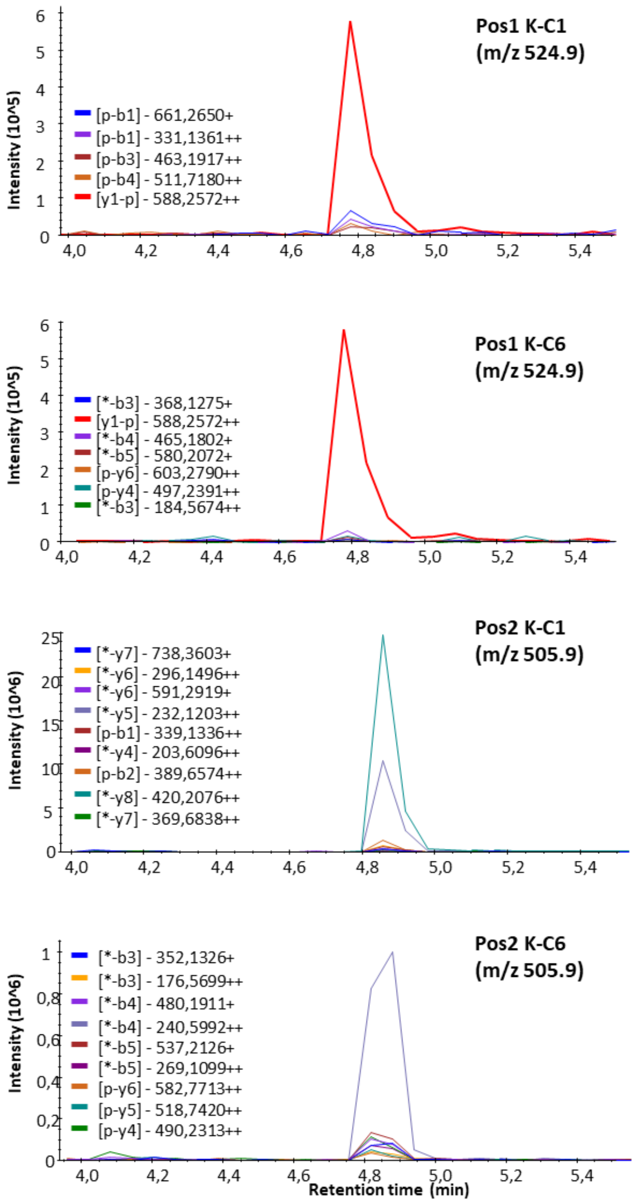

Fig. S14: Targeted LC-MS analysis of possible NOS-bridge peptides in WRKY25.
(A) In the amino acid sequence of the WRKY25 protein the DNA-binding WRKY domains are underlined and the possible positions of the NOS bridge between the (LYS (K) and the Cys (C) are shown in either solid (position 1) or dashed lines (position 2). **(B)** Proteolytic *in-silico* digest with Trypsin (cleavage sides after arginine (R) and lysine (K)) results in 8 different possible peptides. **(C)** Mass signals for transitions of the no bridge peptides SYFK, CTYPDCVSK, SYYK and CTFQGCGVK could be seen in a tryptic in-solution digest of the WRKY25 protein under non-reducing conditions. **(D)** In the same sample digest mass signals for all four of the possible NOS bridge peptide combinations (NOS bridge K-C1 and KC6 in both position 1 and 2) could also be measured.

Table S1: Primer sequences used for different experiments for transactiviation assays

**Primer**	**Sequence**	**Purpose**
PW18 3kb-F	CACCATCTCTTGTTAACAATATCCAA	Amplification Prom. WRKY18 3kb
PW18 3kb-R	AAAAGAAACCTTTATCTTAAGA	Amplification Prom. WRKY18 3kb
PW53 2,8 kb-F	CACCGTTTGGCATTTTCCTACTTTAC	Amplification Prom. WRKY53 2.8 kb
PW53 2,8kb-R	TTTTAGTATATGATTCCCAAAATAG	Amplification Prom. WRKY18 2.8 kb
pBGWFS7_seq_F	CGTTGCGGTTCTGTCAGTTC	Sequencing reporter
pBGWFS7_seq_R	CGTTTACGTCGCCGTCCA	Sequencing reporter
W18-F	CACCATGGACGGTTCTTCGTTTCTCGACATCTC T	Amplification WRKY18
W18-R	TCAATGGTGATGGTGATGATGTGTTCTAGATT GCTCCATTAACC	Amplification WRKY18
W25-F	CACCATGTCTTCCACTTCTTTCACCGACCTTCT TGG	Amplification WRKY25
W25-R	TCAATGGTGATGGTGATGATGCGAGCGACGTA GCGCGGTTG	Amplification WRKY25
W53-F	CACCATGGAAGGAAGAGATATGTTAAGTTGGG AG	Amplification WRKY53
W53-R	TCAATGGTGATGGTGATGATGATAATAAATCG ACTCGTGTAAAA	Amplification WRKY53
pJAN33 Sequ. F	ATCCGACTACAAAGACCA	Sequencing effector
BsaI mutagenesis primer W18 Forward	GAAGAAGGAAGTCTCAGTTTTGG	Mutagenesis of natural BsaI restriction site GAG (Glu) → GAA (Glu)
BsaI mutagenesis primer W18 Reverse	CCAAAACTGAGACTTCCTTCTTC	Mutagenesis of natural BsaI restriction site GAG (Glu) → GAA (Glu)
BsaI mutagenesis primer W25 Forward	TCACCAAGCGACCATCTCAAT	Mutagenesis of natural BsaI restriction site AGA (Arg) → CGA (Arg)
BsaI mutagenesis primer W25 Reverse	ATTGAGATGGTCGCTTGGTGA	Mutagenesis of natural BsaI restriction site AGA (Arg) → CGA (Arg)
W25_Cys17_mut_for	CGTTGACTCTTACGAAGA	Mutagenesis of Cys 17 UGC (Cys) → UCC (Ser)

W25_Cys17_mut_rev	TCGTAAGAGTCAACGCCG	Mutagenesis of Cys 17<br>UGC (Cys) → UCC (Ser)
W25N*_Forward	TTG GGTCTCA CACC ATGTCTTCCACTTCTTTCACCGACCTTC	Amplification of W25N*
W25N*_Reverse	TAA GGTCTC T CGCC ATTAGAGTTCCTACTCACCATGTA	Amplification of W25N*
W25_ΔpD1_55AA_Reverese_1	TAA GGTCTC T CGCC GTTTTGTGTTGCAATCTCATTAG	Amplification of the N-terminal fragment for W25ΔpD1
W25_ΔpD1_55AA_Forward_2	TTT GGTCTC T GGCG AAGAAGATTGTTGAGACGGCTTCTG	Amplification of the C-terminal fragment for W25ΔpD1
W25_ΔpD1_55AA_Reverse_2	TCA GGTCTC T CCTT TCACGAGCGACGTAGCGCGGTT	Amplification of the C-terminal fragment for W25ΔpD1
W25_ΔD2_66AA_ Forward_1	TTG GGTCTC A CACC ATGTCTTCCACTTCTTTCACCGACCTTC	Amplification of the N-terminal fragment for W25ΔD2
W25_ΔD2_66AA_ Reverse_1	GGTCTC T CGCC CTCTTTAACTCCTTTGCTTACTTC	Amplification of the N-terminal fragment for W25ΔD2
W25_ΔD2_66AA_ Forward_2	GGTCTC T GGCG GGAAGACACAATCACGATAT	Amplification of the C-terminal fragment for W25ΔD2
W25_ΔD2_66AA_ Reverse_2	TCA GGTCTC T CCTT TCACGAGCGACGTAGCGCGGTT	Amplification of the C-terminal fragment for W25ΔD2
BsaI Primer W18 N-Terminus Forward	TTG GGTCTC A CACC ATGGACGGTTCTTCGTTTCTCGACA	Amplification of W18N-fragment<br>Creation of BsaI restriction site
BsaI Primer W18 N-Terminus Reverse	AAG GGTCTC A TGGT TGTAGCATCCCCTTCAGAAGCAT	Amplification of W18N-fragment Creation of BsaI restriction site
BsaI Primer W25 C-Terminus Forward	TC ACCA AGAGACCATCTCAATCTTCA	Amplification of W25C-fragment<br>Creation of BsaI restriction site
BsaI Primer W25 C-Terminus Reverse	TCA GGTCTC T CCTT TCACGAGCGACGTAGCGCGGTT	Amplification of W25C-fragment<br>Creation of BsaI restriction site
BsaI Primer W25 N-Terminus Forward	TTG GGTCTC A CACC ATGTCTTCCACTTCTTTCACCGACCTTC	Amplification of W25N-fragment and N-terminal fragment for W25ΔpD1<br>Creation of BsaI restriction site
BsaI Primer W25 N-Terminus Reverse	TGAAGATTGAGAT GGTCTC T TGGT GAA	Amplification of W25N-fragment<br>Creation of BsaI restriction site
BsaI Primer W18 C-Terminus Forward	GCT GGTCTC T ACCA CTACTGAAACATCGGACACAAG	Amplification of W18C-fragment<br>Creation of BsaI restriction site

BsaI Primer W18 C-Terminus Reverse	GAA GGTCTC G CCTT TCATGTTCTAGATTGCTCCATTAAC	Amplification of W18C-fragment<br>Creation of BsaI restriction site
M13 Forward sequencing primer	GTAAAACGACGGCCAG	Sequencing
M13 Reverse sequencing primer	CAGGAAACAGCTATGAC	Sequencing

Table S2: Primers used for cloning BiFC constructs.

Primer	Sequence	Purpose
WRKY18_attB1_F	GGGG ACA AGT TTG TAC AAA AAA GCA GGC TTAATGGACGGTTCTTCGTTTCTCGACA	Amplification WRKY18 and W18N
WRKY18_attB4_R	GGGG ACAACTTTGTATAGAAAAGTTGGGT TCATGTTCTAGATTGCTCCATTAAC	Amplification WRKY18 and W18C
WRKY18_attB3_F	GGGGACA ACT TTG TAT AAT AAA GTT GGAATGGACGGTTCTTCGTTTCTCGAC	Amplification WRKY18 and W18N
WRKY18_attB2_R	GGGG ACCACTTTGTACAAGAAAGCTGGGT TCATGTTCTAGATTGCTCCATTA	Amplification WRKY18 and W18C
WRKY25_attB1_F	GGGG ACA AGT TTG TAC AAA AAA GCA GGC TTA ATGTCTTCCACTTCTTTCACCGAC	Amplification WRKY25 and W25N*, W25ΔpD1,W25ΔD2, W25N
WRKY25_attB4_R	GGGG ACAACTTTGTATAGAAAAGTTGGGT TCACGAGCGACGTAGCGCGGTTG	Amplification WRKY25 and W25N*, W25ΔpD1,W25ΔD2, W25C
WRKY25_attB3_F	GGGG ACA ACT TTG TAT AAT AAA GTT GGA ATGTCTTCCACTTCTTTCACCGAC	Amplification WRKY25 and W25N*, W25ΔpD1,W25ΔD2, W25N
WRKY25_attB2_R	GGGG ACCACTTTGTACAAGAAAGCTGGGT TCACGAGCGACGTAGCGCGGTTG	Amplification WRKY25 and W25ΔpD1,W25ΔD2, W25C
WRKY53_attB1_F	GGGG ACA AGT TTG TAC AAA AAA GCA GGC TTA ATGATGGAAGGAAGAGATATGTTAAGTT	Amplification WRKY53
WRKY53_attB4_R	GGGG ACAACTTTGTATAGAAAAGTTGGGT TTAATAATAAATCGACTCGTGTAAAA	Amplification WRKY53
WRKY53_attB3_F	GGGG ACA ACT TTG TAT AAT AAA GTT GGA ATGATGGAAGGAAGAGATATGTTAAGTT	Amplification WRKY53
WRKY53_attB2_R	GGGG ACCACTTTGTACAAGAAAGCTGGGT TTAATAATAAATCGACTCGTGTAAAA	Amplification WRKY53
M13-F	GTAAAACGACGGCCAG	Sequencing
M13-R	CAGGAAACAGCTATGAC	Sequencing

Table S3: Primers sequences used to clone and create the complementation lines

Primer	Sequence	Purpose
WRKY25_FW_GG	AACAGGTCTCAGGCTCAATGTCTTCC ACTTCTTTCACCGA	Amplification W25 and W25N*, W25ΔpD1, W25ΔD2,W25N
WRKY25_RV_GG	AACAGGTCTCACTGACGAGCGACGT AGCGCGGTTGGGATAT	Amplification W25 and W25ΔpD1, W25ΔD2,W25C
WRKY18_FW_GG	AACAGGTCTCAGGCTCAATGGACGG TTCTTCGTTTCTCG	Amplification W18 and W18N
WRKY18_RV_GG	AACAGGTCTCACTGATGTTCTAGATT GCTCCATTAACCT	Amplification W18 and W18C
EGFP_FW_B_1GG	AACAGGTCTCAAACAATGGTGAGCA AGGGCGAGGAGC	Amplification GFP
EGFP_RV_Cr_1GG	AACAGGTCTCATGCCCTTGTACAGCT CGTCCATGCC	Amplification GFP
GGseq F	TCATTAGGCACCCCAGGCTT	Sequencing
GGseq R	TCTTCGCTATTACGCCAGCT	Sequencing
Z03_F	TGT GGT GTA ACG TTG GAT CTG G	Sequencing
Z03_R	AACTAGGCTCGGACGAAGTAAGC	Sequencing

Table S4: Primers used for qRT-PCR analyses

Primer	Sequence	Purpose
ACTIN	AAGCTCTCCTTTGTTGCTGTT	qRT_PCR foward
ACTIN	GTTGTCTCGTGGATTCCAGCAGCTT	qRT_PCR reverse
WRKY53	CAGACGGGGATGCTACGG	qRT_PCR foward
WRKY53	GGCGAGGCTAATGGTGGT	qRT_PCR reverse

Table S5: Peptide description of LC-MS analyses

peptide	Q1 (m/z)	Q3 (m/z)	CE (Volts)
SYFK pos1 +2 F[y2] +1	272.642	294.181	15
SYFK pos1 +2 Y[y3] +1	272.642	457.245	15
SYYK pos2 +2 Y[y2] +1	280.639	310.176	15
SYYK pos2 +2 Y[y3) +1	280.639	473.239	15
CTYPDCVSK pos1 +2 P[y6] +1	508.215	648.302	23.9
CTYPDCVSK pos1 +2 Y[y7] +1	508.215	811.365	23.9
CTFQGCGVK pos2 +2 Q[y6] +1	471.712	591.292	22.1
CTFQGCGVK pos2 +2 F[y7] +1	471.712	738.36	22.1
SYFK-CTYPDCVSK-pos1K-C1 +3[p-b1]C+2	524.895	331.136	23.2
SYFK-CTYPDCVSK-pos1K-C1 +3[p-b1]C+1	524.895	661.265	23.2
SYFK-CTYPDCVSK-pos1K-C1 +3[p-b3]Y+2	524.895	463.191	23.2
SYFK-CTYPDCVSK-pos1K-C1 +3[p-b4]P+2	524.895	511.718	23.2
SYFK-CTYPDCVSK-pos1K-C1 +3 K[y1-p]+2	524.895	588.257	23.2
SYFK-CTYPDCVSK-pos1K-C6 +3[p-y4]C+2	524.895	497.239	23.2
SYFK-CTYPDCVSK-pos1K-C6 +3[*-b3]Y+1	524.895	368.127	23.2
SYFK-CTYPDCVSK-pos1K-C6 +3[*-b3]Y+2	524.895	184.567	23.2
SYFK-CTYPDCVSK-pos1K-C6 +3 K[y1-p]+2	524.895	588.257	23.2
SYFK-CTYPDCVSK-pos1K-C6 +3[*-b4]P+2	524.895	465.18	23.2
SYFK-CTYPDCVSK-pos1K-C6 +3[*-b5]D+1	524.895	580.207	23.2
SYFK-CTYPDCVSK-pos1K-C6 +3[p-y6]P+2	524.895	603.279	23.2
SYYK-CTFQGCGVK-pos2K-C1 +3[*-y8]T+2	505.892	420.208	22.3
SYYK-CTFQGCGVK-pos2K-C1 +3[*-y7]F+1	505.892	738.36	22.3

SYYK-CTFQGCGVK-pos2K-C1 +3[*-y7]F+2	505.892	369.684	22.3
SYYK-CTFQGCGVK-pos2K-C1 +3[*-y6]Q+2	505.892	296.15	22.3
SYYK-CTFQGCGVK-pos2K-C1 +3[*-y6]Q+	505.892	591.292	22.3
SYYK-CTFQGCGVK-pos2K-C1 +3[*-y5]G+2	505.892	232.12	22.3
SYYK-CTFQGCGVK-pos2K-C1 +3[*-y4]C+2	505.892	203.61	22.3
SYYK-CTFQGCGVK-pos2K-C1 +3[p-b2]T+2	505.892	389.657	22.3
SYYK-CTFQGCGVK-pos2K-C1 +3[p-b1]C+2	505.892	339.134	22.3
SYYK-CTFQGCGVK-pos2K-C6 +3[*-b3]F+1	505.892	352.133	22.3
SYYK-CTFQGCGVK-pos2K-C6 +3[*-b3]F+2	505.892	176.57	22.3
SYYK-CTFQGCGVK-pos2K-C6 +3[*-b4]Q+1	505.892	480.191	22.3
SYYK-CTFQGCGVK-pos2K-C6 +3[*-b4]Q+2	505.892	240.599	22.3
SYYK-CTFQGCGVK-pos2K-C6 +3[*-b5]G+1	505.892	537.213	22.3
SYYK-CTFQGCGVK-pos2K-C6 +3[*-b5]G+2	505.892	269.11	22.3
SYYK-CTFQGCGVK-pos2K-C6 +3[p-y6]Q+2	505.892	582.771	22.3
SYYK-CTFQGCGVK-pos2K-C6 +3[p-y5]G+2	505.892	518.742	22.3
SYYK-CTFQGCGVK-pos2K-C6 +3[p-y4]C+2	505.892	490.231	22.3
Trypsin autolysis peptide 1	421.75	472.3	20
Trypsin autolysis peptide 2	421.75	571.4	20
